# Robust Inference after Random Projections via Hellinger Distance for Location-Scale Family

**DOI:** 10.3390/e21040348

**Published:** 2019-03-29

**Authors:** Lei Li, Anand N. Vidyashankar, Guoqing Diao, Ejaz Ahmed

**Affiliations:** 1 Department of Statistics, George Mason University, Fairfax, VA 22030, USA; 2 Department of Mathematics and Statistics, Brock University, St. Catharines, ON L2S 3A1, Canada

**Keywords:** compressed data, Hellinger distance, representation formula, iterated limits, influence function, consistency, asymptotic normality, location-scale family

## Abstract

Big data and streaming data are encountered in a variety of contemporary applications in business and industry. In such cases, it is common to use random projections to reduce the dimension of the data yielding compressed data. These data however possess various anomalies such as heterogeneity, outliers, and round-off errors which are hard to detect due to volume and processing challenges. This paper describes a new robust and efficient methodology, using Hellinger distance, to analyze the compressed data. Using large sample methods and numerical experiments, it is demonstrated that a routine use of robust estimation procedure is feasible. The role of double limits in understanding the efficiency and robustness is brought out, which is of independent interest.

## 1. Introduction

Streaming data are commonly encountered in several business and industrial applications leading to the so-called Big Data. These are commonly characterized using four V’s: velocity, volume, variety, and veracity. Velocity refers to the speed of data processing while volume refers to the amount of data. Variety refers to various types of data while veracity refers to uncertainty and imprecision in data. It is believed that veracity is due to data inconsistencies, incompleteness, and approximations. Whatever be the real cause, it is hard to identify and pre-process data for veracity in a big data setting. The issues are even more complicated when the data are streaming.

A consequence of the data veracity is that statistical assumptions used for analytics tend to be inaccurate. Specifically, considerations such as model misspecification, statistical efficiency, robustness, and uncertainty assessment-which are standard part of a statistical toolkit-cannot be routinely carried out due to storage limitations. Statistical methods that facilitate simultaneous addressal of twin problems of volume and veracity would enhance the value of the big data. While health care industry and financial industries would be the prime benefactors of this technology, the methods can be routinely applied in a variety of problems that use big data for decision making.

We consider a collection of *n* (*n* is of the order of at least $10^{6}$) observations, assumed to be independent and identically distributed (i.i.d.), from a probability distribution f(·) belonging to a location-scale family; that is,

$$f\left(x;\mu ,\sigma \right)=\frac{1}{\sigma }f\left(\frac{x-\mu }{\sigma }\right),\;\mu \in I\;R,\;\sigma >0.$$

We denote by Θ the parameter space and without loss of generality take it as compact since otherwise it can be re-parametrized in a such a way that the resulting parameter space is compact (see [1]).

The purpose of this paper is to describe a methodology for joint robust and efficient estimation of μ and $\sigma^{2}$ that takes into account (i) storage issues, (ii) potential model misspecifications, and (iii) presence of aberrant outliers. These issues-which are more likely to occur when dealing with massive amounts of data-if not appropriately accounted in the methodological development, can lead to inaccurate inference and misleading conclusions. On the other hand, incorporating them in the existing methodology may not be feasible due to a computational burden.

Hellinger distance-based methods have long been used to handle the dual issue of robustness and statistical efficiency. Since the work of [1,2] statistical methods that invoke alternative objective functions which converge to the objective function under the posited model have been developed and the methods have been shown to possess efficiency and robustness. However, their routine use in the context of big data problems is not feasible due to the complexity in the computations and other statistical challenges. Recently, a class of algorithms-referred to as *Divide and Conquer*—have been developed to address some of these issues in the context of likelihood. These algorithms consist in distributing the data across multiple processors and, in the context of the problem under consideration, estimating the parameters from each processor separately and then combining them to obtain an overall estimate. The algorithm assumes availability of several processors, *with substantial processing power,* to solve the complex problem at hand. Since robust procedures involve complex iterative computations-invoking the increased demand for several high-speed processors and enhanced memory-routine use of available analytical methods in a big data setting is challenging. Maximum likelihood method of estimation in the context of location-scale family of distributions has received much attention in the literature ([3,4,5,6,7]). It is well-known that the maximum likelihood estimators (MLE) of location-scale families may not exist unless the defining function f(·) satisfies certain regularity conditions. Hence, it is natural to ask if other methods of estimation such as minimum Hellinger distance estimator(MHDE) under weaker regularity conditions. This manuscript provides a first step towards addressing this question. Random projections and sparse random projections are being increasingly used to “compress data” and then use the resulting compressed data for inference. The methodology, primarily developed by computer scientists, is increasingly gaining attention among the statistical community and is investigated in a variety of recent work ([8,9,10,11,12]). In this manuscript, we describe a Hellinger distance-based methodology for robust and efficient estimation after the use of random projections for compressing i.i.d data belonging to the location-scale family. The proposed method consists in reducing the dimension of the data to facilitate the ease of computations and simultaneously maintain robustness and efficiency when the posited model is correct. While primarily developed to handle big and streaming data, the approach can also be used to handle privacy issues in a variety of applications [13].

The rest of the paper is organized as follows: Section 2 provides background on minimum Hellinger distance estimation; Section 3 is concerned with the development of Hellinger distance-based methods for compressed data obtained after using random projections; additionally, it contains the main results and their proofs. Section 4 contains results of the numerical experiments and also describes an algorithm for implementation of the proposed methods. Section 5 contains a real data example from financial analytics. Section 6 is concerned with discussions and extensions. Section 7 contains some concluding remarks.

## 2. Background on Minimum Hellinger Distance Estimation

Ref. [1] proposed minimum Hellinger distance (MHD) estimation for i.i.d. observations and established that MHD estimators (MHDE) are simultaneously robust and first-order efficient under the true model. Other researchers have investigated related estimators, for example, [14,15,16,17,18,19,20]. These authors establish that when the model is correct, the MHDE is asymptotically equivalent to the maximum likelihood estimator (MLE) in a variety of independent and dependent data settings. For a comprehensive discussion of minimum divergence theory see [21].

We begin by recalling that the Hellinger distance between two probability densities is the $L^{2}$ distance between the square root of the densities. Specifically, let, for p≥1, $\left|\right|\cdot {\left|\right|}_{p}$ denote the $L^{p}$ norm defined by

$$\begin{array}{c} {\left||h|\right|}_{p}={\left\{\int {\left|h\right|}^{p}\right\}}^{1/p}. \end{array}$$

The Hellinger distance between the densities f(·) and g(·) is given by

$$\begin{array}{c} H^{2}\left(f\left(\cdot \right),g\left(\cdot \right)\right)=\left|\right|f^{1/2}\left(\cdot \right)-g^{1/2}\left(\cdot \right){\left|\right|}_{2}^{2}. \end{array}$$

Let f(·|θ) denote the density of $I\;R^{d}$ valued independent and identically distributed random variables $X_{1},\cdots ,X_{n}$, where $\theta \in \Theta \subset I\;R^{p}$; let $g_{n}\left(\cdot \right)$ be a nonparametric density estimate (typically a kernel density estimator). The Hellinger distance between f(·|θ) and $g_{n}\left(\cdot \right)$ is then

$$\begin{array}{c} H^{2}\left(f\left(\cdot |\theta \right),g_{n}\left(\cdot \right)\right)=\left|\right|f^{1/2}\left(\cdot |\theta \right)-g_{n}^{1/2}\left(\cdot \right){\left|\right|}_{2}^{2}. \end{array}$$

The MHDE is a mapping T(·) from the set of all densities to $I\;R^{p}$ defined as follows:

(1) $$\begin{array}{c} \theta_{g}=T\left(g\right)=\underset{\theta \in \Theta }{\mathrm{argmin}}\;H^{2}\left(f(\cdot |\theta ),g(\cdot )\right). \end{array}$$

Please note that the above minimization problem is equivalent to maximizing $A\left(f(\cdot |\theta ),g(\cdot )\right)=\int f^{1/2}\left(x|\theta \right)g^{1/2}\left(x\right)dx$. Hence MHDE can alternatively be defined as

$$\begin{array}{c} \theta_{g}=\underset{\theta \in \Theta }{\mathrm{argmax}}\;A\left(f(\cdot |\theta ),g(\cdot )\right). \end{array}$$

To study the robustness of MHDE, ref. [1] showed that to assess the robustness of a functional with respect to the gross-error model it is necessary to examine the α-influence curve rather than the influence curve, except when the influence curve provides a uniform approximation to the α-influence curve. Specifically, the α-influence function (${\mathrm{IF}}_{\alpha }\left(\theta ,z\right)$) is defined as follows: for θ∈Θ, let $f_{\alpha ,\theta ,z}=\left(1-\alpha \right)f\left(\cdot |\theta \right)+\alpha \eta_{z}$, where $\eta_{z}$ denotes the uniform density on the interval (z−ϵ,z+ϵ), where ϵ>0 is small, α∈(0,1), z∈IR; the α-influence function is then defined to be

(2) $$\begin{array}{c} {\mathrm{IF}}_{\alpha }\left(\theta ,z\right)=\frac{T(f_{\alpha ,\theta ,z})-\theta }{\alpha }, \end{array}$$

where $T(f_{\alpha ,\theta ,z})$ is the functional for the model with density $f_{\alpha ,\theta ,z}\left(\cdot \right)$. Equation (2) represents a complete description of the behavior of the estimator in the presence of contamination, up to the shape of the contaminating density. If ${\mathrm{IF}}_{\alpha }\left(\theta ,z\right)$ is a bounded function of *z* such that $\lim_{z\to \infty }{\mathrm{IF}}_{\alpha }\left(\theta ,z\right)=0,\mathrm{for}\;\mathrm{every}\;\theta \in \Theta$, then the functional *T* is robust at f(·|θ) against 100%α contamination by gross errors at arbitrary large value *z*. The influence function can be obtained by letting α→0. Under standard regularity conditions, the minimum divergence estimators (MDE) are first order efficient and have the same influence function as the MLE under the model, which is often unbounded. Hence the robustness of these estimators cannot be explained through their influence functions. In contrast, the α-influence function of the estimators are often bounded, continuous functions of the contaminating point. Finally, this approach often leads to high breakdown points in parametric estimation. Other explanations can also be found in [22,23].

Ref. [1] showed that the MHDE of location has a breakdown point equal to 50%. Roughly speaking, the breakdown point is the smallest fraction of data that, when strategically placed, can cause an estimator to take arbitrary values. Ref. [24] obtained breakdown results for MHDE of multivariate location and covariance. They showed that the affine-invariant MHDE for multivariate location and covariance has a breakdown point of at least 25%. Ref. [18] showed that the MHDE has 50% breakdown in some discrete models.

## 3. Hellinger Distance Methodology for Compressed Data

In this section we describe the Hellinger distance-based methodology as applied to the compressed data. Since we are seeking to model the streaming independent and identically distributed data, we denote by *J* the number of observations in a fixed time-interval (for instance, every ten minutes, or every half-hour, or every three hours). Let *B* denote the total number of time intervals. Alternatively, *B* could also represent the number of sources from which the data are collected. Then, the incoming data can be expressed as $\left\{X_{jl},1\leq j\leq J;1\leq l\leq B\right\}$. Throughout this paper, we assume that the density of $X_{jl}$ belongs to a location-scale family and is given by $f\left(x;\theta^{*}\right)=\frac{1}{\sigma^{*}}f\left(\frac{x-\mu^{*}}{\sigma^{*}}\right)$, where $\theta^{*}=\left(\mu^{*},\sigma^{*}\right)$. A typical example is a data store receiving data from multiple sources, for instance financial or healthcare organizations, where information from multiple sources across several hours are used to monitor events of interest such as cumulative usage of certain financial instruments or drugs.

### 3.1. Random Projections

Let $R_{l}=\left(r_{ijl}\right)$ be a S×J matrix, where *S* is the number of compressed observations in each time interval, S≪J, and $r_{ijl}$’s are independent and identically distributed random variables and assumed to be independent of $\left\{X_{jl},j=1,2,\cdots ,J;1\leq l\leq B\right\}$. Let

$$\begin{array}{c} {\tilde{Y}}_{il}=\sum_{j=1}^{J}r_{ijl}X_{jl} \end{array}$$

and set $\tilde{Y_{l}}={\left({\tilde{Y}}_{1l},\cdots ,{\tilde{Y}}_{Sl}\right)}^{\prime }$; in matrix form this can be expressed as ${\tilde{Y}}_{l}=R_{l}X_{l}$. The matrix $R_{l}$ is referred to as the *sensing matrix* and $\left\{{\tilde{Y}}_{il},i=1,2\cdots ,S;l=1,2,\cdots ,B\right\}$ is referred to as the *compressed data*. The total number of compressed observations m=SB is much smaller than the number of original observations n=JB. We notice here that $R_{l}$’s are independent and identically distributed random matrices of order S×J. Referring to each time interval or a source as a group, the following Table 1 is a tabular representation of the compressed data.

In random projections literature, the distribution of $r_{ijl}$ is typically taken to be Gaussian; but other distributions such as Rademacher distribution, exponential distribution and extreme value distributions are also used (for instance, see [25]). In this paper, we do not make any strong distributional assumptions on $r_{ijl}$. We only assume that $E\left[r_{ijl}\right]=1$ and $\mathrm{Var}\left[r_{ijl}\right]=\gamma_{0}^{2}$, where E[·] represents the expectation of the random variable and $\mathrm{Var}\left[\cdot \right]$ represents the variance of the random variable. Additionally, we denote the density of $r_{ijl}$ by q(·).

We next return to the storage issue. When S=1 and $r_{ijl}=1$, ${\tilde{Y}}_{il}$ is a sum of *J* random variables. In this case, one retains (stores) only the sum of *J* observations and robust estimates of $\theta^{*}$ are sought using the sum of observations. In other situations, that is when $r_{ijl}$ are not degenerate at 1, the distribution of ${\tilde{Y}}_{il}$ is complicated. Indeed, even if $r_{ijl}$ are assumed to be normally distributed, the marginal distribution of ${\tilde{Y}}_{il}$ is complicated. The conditional distribution is ${\tilde{Y}}_{il}$ (given $r_{ijl}$) is a weighted sum of location scale distributions and does not have a useful closed form expression. Hence, in general for these problems the MLE method is *not feasible*. We denote by $\omega_{il}^{2}=\sum_{j=1}^{J}r_{ijl}^{2}$ and work with the random variables $Y_{il}\equiv \omega_{il}^{-1}{\tilde{Y}}_{il}.$ We denote the true density of $Y_{il}$ to be $h_{J}\left(\cdot |\theta^{*},\gamma_{0}\right)$. Also, when $\gamma_{0}=0$ (which implies $r_{ijl}\equiv 1$) we denote the true density of $Y_{il}$ by $h^{*J}\left(\cdot |\theta^{*}\right)$ to emphasize that the true density is a convolution of *J* independent and identically distributed random variables.

### 3.2. Hellinger Distance Method for Compressed Data

In this section, we describe the Hellinger distance-based method for estimating the parameters of the location scale family using the compressed data. As described in the last section, let $\left\{X_{jl},j=1,2,\cdots ,J;l=1,2,\cdots ,B\right\}$ be a doubly indexed collection of independent and identically distributed random variables with true density $\frac{1}{\sigma^{*}}f\left(\frac{\cdot -\mu^{*}}{\sigma^{*}}\right)$. Our goal is to estimate $\theta^{*}=\left(\mu^{*},\sigma^{2*}\right)$ using the compressed data $\left\{Y_{il},i=1,2,\cdots ,S;l=1,2,\cdots ,B\right\}$. We re-emphasize here that the density of $Y_{il}$ depends additionally on $\gamma_{0}$, the variance of the sensing random variables $r_{ijl}$.

To formulate the Hellinger-distance estimation method, let G be a class of densities metrized by the $L_{1}$ distance. Let $\left\{h_{J}\left(\cdot |\theta ,\gamma_{0}\right);\theta \in \Theta \right\}$ be a parametric family of densities. The Hellinger distance functional *T* is a measurable mapping mapping from G to Θ, defined as follows:

$$\begin{array}{ccc} T(g) & \equiv & \underset{\theta }{arg min}\int_{I\;R}{\left(g^{\frac{1}{2}}\left(y\right)-h_{J}^{\frac{1}{2}}\left(y|\theta ,\gamma_{0}\right)\right)}^{2}dy\\ & = & \underset{\theta }{arg min}HD^{2}\left(g,h_{J}\left(\cdot |\theta ,\gamma_{0}\right)\right)=\theta_{g}^{*}\left(\gamma_{0}\right). \end{array}$$

When $g\left(\cdot \right)=h_{J}\left(\cdot |\theta^{*},\gamma_{0}\right)$, then under additional assumptions $\theta_{g}^{*}\left(\gamma_{0}\right)=\theta^{*}\left(\gamma_{0}\right)$. Since minimizing the Hellinger-distance is equivalent to maximizing the affinity, it follows that

$$\begin{array}{c} T\left(g\right)=\underset{\theta }{arg max}A\left(g,h_{J}\left(\cdot |\theta ,\gamma_{0}\right)\right),\;\mathrm{where} \end{array}$$

$$\begin{array}{c} A\left(g,h_{J}\left(\cdot |\theta ,\gamma_{0}\right)\right)\equiv \int_{I\;R}g^{\frac{1}{2}}\left(y\right)h_{J}^{\frac{1}{2}}\left(y|\theta ,\gamma_{0}\right)dy. \end{array}$$

It is worth noticing here that

(3) $$\begin{array}{c} A\left(g,h_{J}\left(\cdot |\theta ,\gamma_{0}\right)\right)=1-\frac{1}{2}HD^{2}\left(g,h_{J}\left(\cdot |\theta ,\gamma_{0}\right)\right). \end{array}$$

To obtain the Hellinger distance estimator of the true unknown parameters $\theta^{*}$, expectedly we choose the parametric family $h_{J}\left(\cdot |\theta ,\gamma_{0}\right)$ to be density of $Y_{il}$ and g(·) to be a non-parametric $L_{1}$ consistent estimator $g_{B}\left(\cdot \right)$ of $h_{J}\left(\cdot |\theta ,\gamma_{0}\right)$. Thus, the MHDE of $\theta_{B}^{*}$ is given by

$$\begin{array}{ccc} {\hat{\theta }}_{B}\left(\gamma_{0}\right) & = & \underset{\theta }{arg max}A\left(g_{B},h_{J}\left(\cdot |\theta ,\gamma_{0}\right)\right)=T\left(g_{B}\right). \end{array}$$

In the notation above, we emphasize the dependence of the estimator on the variance of the projecting random variables. We notice here that the solution to (1) may not be unique. In such cases, we choose one of the solutions in a measurable manner.

The choice of the density estimate, typically employed in the literature is the kernel density estimate. However, in the setting of the compressed data investigated here, there are *S* observations per group. These *S* observations are, conditioned on $r_{ijl}$ independent; however they are marginally dependent (if S>1). In the case when S>1, we propose the following formula for $g_{B}\left(\cdot \right)$. First, we consider the estimator

$$\begin{array}{c} g_{B}^{\left(i\right)}\left(y\right)=\frac{1}{Bc_{B}}\sum_{l=1}^{B}K\left(\frac{y-Y_{il}}{c_{B}}\right),\;i=1,2,\cdots ,S. \end{array}$$

With this choice, the MHDE of $\theta_{B}^{*}$ is given by, for 1≤i≤S,

(4) $$\begin{array}{c} {\hat{\theta }}_{i,B}\left(\gamma_{0}\right)=\underset{\theta }{arg max}A\left(g_{B}^{\left(i\right)},h_{J}\left(\cdot |\theta ,\gamma_{0}\right)\right). \end{array}$$

The above estimate of the density chooses $i^{th}$ observation from each group and obtains the kernel density estimator using the *B* independent and identically distributed compressed observations. This is one choice for the estimator. Of course, alternatively, one could obtain $S^{B}$ different estimators by choosing different combinations of observations from each group.

It is well-known that the estimator is almost surely $L_{1}$ consistent for $h_{J}\left(\cdot |\theta^{*},\gamma_{0}\right)$ as long as $c_{B}\to 0$ and $Bc_{B}\to \infty$ as B→∞. Hence, under additional regularity and identifiability conditions and further conditions on the bandwidth $c_{B}$, existence, uniqueness, consistency and asymptotic normality of ${\hat{\theta }}_{i,B}\left(\gamma_{0}\right)$, for fixed $\gamma_{0}$, follows from the existing results in the literature.

When $\gamma_{0}=0$ and $r_{ijl}\equiv 1$, as explained previously, the true density is a J–fold convolution of $f(\cdot |\theta^{*})$, it is natural to ask the following question: if one lets $\gamma_{0}\to 0$, will the asymptotic results converge to what one would obtain by taking $\gamma_{0}=0$. We refer to this property as a *continuity property in* $\gamma_{0}$ of the procedure. Furthermore, it is natural to wonder if these asymptotic properties can be established uniformly in $\gamma_{0}$. If that is the case, then one can also allow $\gamma_{0}$ to depend on *B*. This idea has an intuitive appeal since one can choose the parameters of the sensing random variables to achieve an optimal inferential scheme. We address some of these issues in the next subsection.

Finally, we emphasize here that while we do not require S>1, in applications involving streaming data and privacy problems *S* tends to greater than one. In problems where the variance of sensing variables are large, one can obtain an overall estimator by averaging ${\hat{\theta }}_{i,B}\left(\gamma_{0}\right)$ over various choices of 1≤i≤S; that is,

(5) $$\begin{array}{c} {\hat{\theta }}_{B}\left(\gamma_{0}\right)=\frac{1}{S}\sum_{i=1}^{S}{\hat{\theta }}_{i,B}\left(\gamma_{0}\right). \end{array}$$

**Figure 1:**
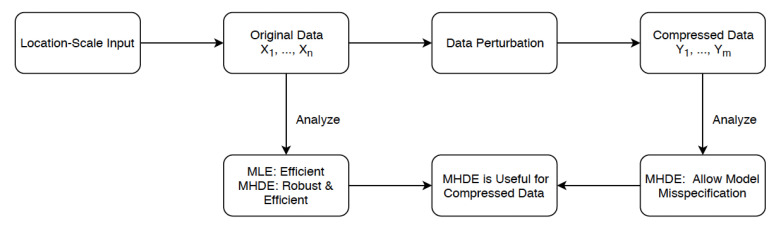
MLE vs. MHDE after Data Compression.

The averaging improves the accuracy of the estimator in small compressed samples (data not presented). For this reason, we provide results for this general case, even though our simulation and theoretical results demonstrate that for some problems considered in this paper, *S* can be taken to be one. We now turn to our main results which are presented in the next subsection. The following Figure 1 provides a overview of our work.

### 3.3. Main Results

In this section we state our main results concerning the asymptotic properties of the MHDE of compressed data $Y_{il}$. We emphasize here that we only store $\{\left({\tilde{Y}}_{il},r_{i\cdot l},\omega_{il}^{2}\right):i=1,2,\cdots ,S;l=1,2,\cdots ,B\}.$ Specifically, we establish the continuity property in $\gamma_{0}$ of the proposed methods by establishing the existence of the iterated limits. This provides a first step in establishing the double limit. The first proposition is well-known and is concerned with the existence and uniqueness of MHDE for the location-scale family defined in () using compressed data.

**Proposition** **1.** *Assume that $h_{J}\left(\cdot |\theta ,\gamma_{0}\right)$ is a continuous density function. Assume further that if $\theta_{1}\neq \theta_{2}$. Then for every $\gamma_{0}\geq 0$, $h_{J}\left(y|\theta_{1},\gamma_{0}\right)\neq h_{J}\left(y|\theta_{2},\gamma_{0}\right)$ on a set of positive Lebesgue measure, the MHDE in (4) exists and is unique.*

**Proof.**  The proof follows from Theorem 2.2 of [20] since, without loss of generality, Θ is taken to be compact and the density function $h_{J}\left(\cdot |\theta ,\gamma_{0}\right)$ is continuous in θ. □

**Consistency**: We next turn our attention to consistency. As explained previously, under regularity conditions for each fixed $\gamma_{0}$, the MHDE ${\hat{\theta }}_{i,B}\left(\gamma_{0}\right)$ is consistent for $\theta^{*}\left(\gamma_{0}\right)$. The next result says that under additional conditions, the consistency property of MHDE is continuous in $\gamma_{0}$.

**Proposition** **2.** *Let $h_{J}\left(\cdot |\theta ,\gamma_{0}\right)$ be a continuous probability density function satisfying the conditions of Proposition 1. Assume that*

(6) $$\begin{array}{c} \lim_{\gamma_{0}\to 0}\underset{\theta \in \Theta }{\sup}\int_{I\;R}\left|h_{J}\left(y|\theta ,\gamma_{0}\right)-h^{*J}\left(y|\theta \right)\right|dy=0. \end{array}$$

Then, with probability one (wp1) the iterated limits also exist and equals $\theta^{*}$; that is, for 1≤i≤S,

$$\begin{array}{c} \lim_{B\to \infty }\lim_{\gamma_{0}\to 0}{\hat{\theta }}_{i,B}\left(\gamma_{0}\right)=\lim_{\gamma_{0}\to 0}\lim_{B\to \infty }{\hat{\theta }}_{i,B}\left(\gamma_{0}\right)=\theta^{*}. \end{array}$$

**Proof.**  Without loss of generality let Θ be compact since otherwise it can be embedded into a compact set as described in [1]. Since f(·) is continuous in θ and g(·) is continuous in $\gamma_{0}$, it follows that $h_{J}\left(\cdot |\theta ,\gamma_{0}\right)$ is continuous in θ and $\gamma_{0}$. Hence by Theorem 1 of [1] for every fixed $\gamma_{0}\geq 0$ and 1≤i≤S,

$$\begin{array}{c} \lim_{B\to \infty }{\hat{\theta }}_{i,B}\left(\gamma_{0}\right)=\theta^{*}\left(\gamma_{0}\right). \end{array}$$

Thus, to verify the convergence of $\theta^{*}\left(\gamma_{0}\right)$ to $\theta^{*}$ as $\gamma_{0}\to 0$, we first establish, using (6), that

$$\begin{array}{c} \lim_{\gamma_{0}\to 0}\underset{\theta \in \Theta }{\sup}\left|A\left(h_{J}\left(\cdot |\theta ,\gamma_{0}\right),h^{*J}\left(\cdot |\theta \right)\right)-1\right|=0. \end{array}$$

To this end, we first notice that

$$\begin{array}{c} \underset{\theta \in \Theta }{\sup}HD^{2}\left(h_{J}\left(\cdot |\theta ,\gamma_{0}\right),h^{*J}\left(\cdot |\theta \right)\right)\leq \underset{\theta \in \Theta }{\sup}\int_{I\;R}|(h_{J}\left(y|\theta ,\gamma_{0}\right)-h^{*J}\left(y|\theta \right)|dy. \end{array}$$

Hence, using (3),

$$\begin{array}{ccc} \underset{\theta \in \Theta }{\sup}\left|A\left(h_{J}\left(\cdot |\theta ,\gamma_{0}\right),h^{*J}\left(\cdot |\theta \right)\right)-1\right| & = & \frac{1}{2}\underset{\theta \in \Theta }{\sup}HD^{2}\left(h_{J}\left(\cdot |\theta ,\gamma_{0}\right),h^{*J}\left(\cdot |\theta \right)\right)\\ & \to & 0\;\;\;\mathrm{as}\;\gamma_{0}\to 0. \end{array}$$

Hence,

$$\begin{array}{c} \lim_{\gamma_{0}\to 0}A\left(h_{J}\left(\cdot |\theta^{*}\left(\gamma_{0}\right),\gamma_{0}\right),h^{*J}\left(\cdot |\theta^{*}\left(\gamma_{0}\right)\right)\right)=1. \end{array}$$

Also, by continuity,

$$\begin{array}{c} \lim_{\gamma_{0}\to 0}A\left(h^{*J}\left(\cdot |\theta^{*}\left(\gamma_{0}\right),\gamma_{0}\right),h^{*J}\left(\cdot |\theta^{*}\right)\right)=1, \end{array}$$

which, in turn implies that

$$\begin{array}{c} \lim_{\gamma_{0}\to 0}A\left(h_{J}\left(\cdot |\theta^{*}\left(\gamma_{0}\right),\gamma_{0}\right),h^{*J}\left(\cdot |\theta^{*}\right)\right)=1. \end{array}$$

Thus existence of the iterated limit first as B→∞ and then $\gamma_{0}\to 0$ follows using compactness of Θ and the identifiability of the model. As for the other iterated limit, again notice notice that for each 1≤i≤S, $A(g_{B}^{\left(i\right)},h_{J}\left(\cdot |\theta ,\gamma_{0}\right))$ converges to $A(g_{B}^{\left(i\right)},h^{*J}\left(\cdot |\theta \right))$ with probability one as $\gamma_{0}$ converges to 0. The result then follows again by an application of Theorem 1 of [20]. □

**Remark** **1.** *Verification of condition (6) seems to be involved even in the case of standard Gaussian random variables and standard Gaussian sensing random variables. Indeed in this case, the density of $h_{J}\left(\cdot |\theta ,\gamma_{0}\right)$ is a J−fold convolution of a Bessel function of second kind. It may be possible to verify the condition (6) using the properties of these functions and compactness of the parameter space ***Θ***. However, if one is focused only on weak-consistency, it is an immediate consequence of Theorems 1 and 2 below and condition (6) is not required. Finally, it is worth mentioning here that the convergence in (6) without uniformity over ***Θ*** is a consequence of convergence in probability of $r_{ijl}$ to 1 and Glick’s Theorem.*

**Asymptotic limit distribution:** We now proceed to investigate the limit distribution of $\theta_{B}^{*}\left(\gamma_{0}\right)$ as B→∞ and $\gamma_{0}\to 0$. It is well-known that for fixed $\gamma_{0}\geq 0$, after centering and scaling, $\theta_{B}^{*}\left(\gamma_{0}\right)$ has a limiting Gaussian distribution, under appropriate regularity conditions (see for instance [20]). However to evaluate the iterated limits as $\gamma_{0}\to 0$ and B→∞, additional refinements of the techniques in [20] are required. To this end, we start with additional notations. Let $s_{J}\left(\cdot |\theta ,\gamma_{0}\right)=h_{J}^{\frac{1}{2}}\left(\cdot |\theta ,\gamma_{0}\right)$ and let the score function be denoted by $u_{J}\left(\cdot |\theta ,\gamma_{0}\right)\equiv \nabla \log h_{J}\left(\cdot |\theta ,\gamma_{0}\right)={\left(\frac{\partial \;\log\;h_{J}\left(\cdot |\theta ,\gamma_{0}\right)}{\partial \mu },\frac{\partial \;\log\;h_{J}\left(\cdot |\theta ,\gamma_{0}\right)}{\partial \sigma }\right)}^{\prime }$. Also, the Fisher information $I(\theta \left(\gamma_{0}\right))$ is given by

$$\begin{array}{c} I\left(\theta \left(\gamma_{0}\right)\right)=\int_{I\;R}u_{J}\left(y|\theta ,\gamma_{0}\right)u_{J}^{\prime }\left(y|\theta ,\gamma_{0}\right)h_{J}\left(y|\theta ,\gamma_{0}\right)dy. \end{array}$$

In addition, let ${\dot{s}}_{J}\left(\cdot |\theta ,\gamma_{0}\right)$ be the gradient of $s_{J}\left(\cdot |\theta ,\gamma_{0}\right)$ with respect to θ, and ${\ddot{s}}_{J}\left(\cdot |\theta ,\gamma_{0}\right)$ is the second derivative matrix of $s_{J}\left(\cdot |\theta ,\gamma_{0}\right)$ with respect to θ. In addition, let $t_{J}\left(\cdot |\theta \right)=h^{*J\frac{1}{2}}\left(\cdot |\theta \right)$ and $v_{J}\left(\cdot |\theta \right)=\nabla \log h^{*J}\left(\cdot |\theta \right)$. Furthermore, let $Y_{il}^{*}$ denote $Y_{il}$ when $\gamma_{0}\equiv 0$. Please note that in this case, $Y_{il}=Y_{1l}$ for all i=1,2,⋯,S. The corresponding kernel density estimate of $Y_{il}^{*}$ is given by

(7) $$\begin{array}{c} g_{B}^{*}\left(y\right)=\frac{1}{Bc_{B}}\sum_{l=1}^{B}K\left(\frac{y-Y_{il}^{*}}{c_{B}}\right). \end{array}$$

We emphasize here that we suppress *i* on the LHS of the above equation since $g_{B}^{(i)*}\left(\cdot \right)$ are equal for all 1≤i≤S.

The iterated limit distribution involves additional regularity conditions which are stated in the Appendix. The first step towards this aim is a representation formula which expresses the quantity of interest, *viz.*, $\sqrt{B}({\hat{\theta }}_{i,B}\left(\gamma_{0}\right)-\theta^{*}\left(\gamma_{0}\right))$ as a sum of two terms, one involving sums of *compressed* i.i.d. random variables and the other involving remainder terms that converge to 0 at a specific rate. This expression will appear in different guises in the rest of the manuscript and will play a critical role in the proofs.

### 3.4. Representation Formula

Before we state the lemma, we first provide two crucial assumptions that allow differentiating the objective function and interchanging the differentiation and integration:

**Model assumptions on**$h_{J}\left(\cdot |\theta ,\gamma_{0}\right)$

(**D1**) $h_{J}\left(\cdot |\theta ,\gamma_{0}\right)$ is twice continuously differentiable in θ.

(**D2**) Assume further that $||\nabla s_{J}\left(\cdot |\theta ,\gamma_{0}\right){\left|\right|}_{2}$ is continuous and bounded.

**Lemma** **1.** *Assume that the conditions (**D1**) and (**D2**) hold. Then for every 1≤i≤S and $\gamma_{0}\geq 0,$ the following holds:*

(8) $$\begin{array}{c} B^{\frac{1}{2}}{\left({\hat{\theta }}_{i,B}\left(\gamma_{0}\right)-\theta^{*}\left(\gamma_{0}\right)\right)}^{\prime }=A_{1B}\left(\gamma_{0}\right)+A_{2B}\left(\gamma_{0}\right),\;\mathrm{where} \end{array}$$

(9) $$\begin{array}{c} A_{1B}\left(\gamma_{0}\right)=B^{\frac{1}{2}}D_{B}^{-1}\left({\tilde{\theta }}_{i,B}\left(\gamma_{0}\right)\right)T_{B}\left(\gamma_{0}\right),\;A_{2B}\left(\gamma_{0}\right)=B^{\frac{1}{2}}D_{B}^{-1}\left({\tilde{\theta }}_{i,B}\left(\gamma_{0}\right)\right)R_{B}\left(\gamma_{0}\right), \end{array}$$

(10) $$\begin{array}{c} {\tilde{\theta }}_{i,B}\left(\gamma_{0}\right)\in U_{B}\left(\theta^{\prime }\left(\gamma_{0}\right)\right),\;U_{B}\left(\theta^{\prime }\left(\gamma_{0}\right)\right)=\left\{\theta^{\prime }:\theta^{\prime }\left(\gamma_{0}\right)=t\theta^{*}\left(\gamma_{0}\right)+\left(1-t\right){\hat{\theta }}_{i,B}\left(\gamma_{0}\right),t\in \left[0,1\right]\right\}, \end{array}$$

(11) $$\begin{array}{ccc} D_{B}\left(\theta \left(\gamma_{0}\right)\right) & = & -\frac{1}{2}\int_{I\;R}{\dot{u}}_{J}\left(y|\theta ,\gamma_{0}\right)s_{J}\left(y|\theta ,\gamma_{0}\right)g_{B}^{\left(i\right)\frac{1}{2}}\left(y\right)dy\\ & \; & -\frac{1}{4}\int_{I\;R}u_{J}\left(y|\theta ,\gamma_{0}\right)u_{J}^{\prime }\left(y|\theta ,\gamma_{0}\right)s_{J}\left(y|\theta ,\gamma_{0}\right)g_{B}^{\left(i\right)\frac{1}{2}}\left(y\right)dy\\ & \equiv & D_{1B}\left(\theta \left(\gamma_{0}\right)\right)+D_{2B}\left(\theta \left(\gamma_{0}\right)\right), \end{array}$$

(12) $$\begin{array}{c} T_{B}\left(\gamma_{0}\right)\equiv \frac{1}{4}\int_{I\;R}u_{J}\left(y|\theta^{*},\gamma_{0}\right)\left(h_{J}\left(y|\theta^{*},\gamma_{0}\right)-g_{B}^{\left(i\right)}\left(y\right)\right)dy,\;\mathrm{and} \end{array}$$

(13) $$\begin{array}{c} R_{B}\left(\gamma_{0}\right)\equiv \frac{1}{4}\int_{I\;R}u_{J}\left(y|\theta^{*},\gamma_{0}\right){\left(h_{J}^{\frac{1}{2}}\left(y|\theta^{*},\gamma_{0}\right)-g_{B}^{\left(i\right)\frac{1}{2}}\left(y\right)\right)}^{2}dy. \end{array}$$

**Proof.**  By algebra, note that ${\dot{s}}_{J}\left(y|\theta ,\gamma_{0}\right)=\frac{1}{2}u_{J}\left(y|\theta ,\gamma_{0}\right)s_{J}\left(y|\theta ,\gamma_{0}\right).$ Furthermore, the second partial derivative of $s_{J}\left(\cdot |\theta ,\gamma_{0}\right)$ is given by ${\ddot{s}}_{J}\left(y|\theta ,\gamma_{0}\right)=\frac{1}{2}{\dot{u}}_{J}\left(y|\theta ,\gamma_{0}\right)s_{J}\left(y|\theta ,\gamma_{0}\right)+\frac{1}{4}u_{J}\left(y|\theta ,\gamma_{0}\right)u_{J}^{\prime }\left(y|\theta ,\gamma_{0}\right)s_{J}\left(y|\theta ,\gamma_{0}\right).$ Now using (**D1**) and (**D2**) and partially differentiating $HD_{B}^{2}\left(\theta (\gamma_{0})\right)\equiv HD^{2}\left(g_{B}^{\left(i\right)}\left(\cdot \right),h_{J}\left(\cdot |\theta ,\gamma_{0}\right)\right)$ with respect to θ and setting it equal to 0, the estimating equations for $\theta^{*}\left(\gamma_{0}\right)$ is

(14) $$\begin{array}{c} \nabla HD_{B}^{2}\left(\theta^{*}\left(\gamma_{0}\right)\right)=0. \end{array}$$

Let ${\hat{\theta }}_{i,B}\left(\gamma_{0}\right)$ be the solution to (14). Now applying first order Taylor expansion of (14) we get

$$\begin{array}{c} \nabla HD_{B}^{2}\left(\theta^{*}\left(\gamma_{0}\right)\right)=\nabla HD_{B}^{2}({\hat{\theta }}_{i,B}\left(\gamma_{0}\right))+D_{B}\left({\tilde{\theta }}_{i,B}\left(\gamma_{0}\right)\right)({\hat{\theta }}_{i,B}\left(\gamma_{0}\right)-\theta^{*}\left(\gamma_{0}\right)), \end{array}$$

where ${\tilde{\theta }}_{i,B}\left(\gamma_{0}\right)$ is defined in (10), and $D_{B}\left(\cdot \right)$ is given by

$$\begin{array}{ccc} D_{B}\left(\theta \left(\gamma_{0}\right)\right) & = & -\frac{1}{2}\int_{I\;R}{\dot{u}}_{J}\left(y|\theta ,\gamma_{0}\right)s_{J}\left(y|\theta ,\gamma_{0}\right)g_{B}^{\left(i\right)\frac{1}{2}}\left(y\right)dy\\ & \; & -\frac{1}{4}\int_{I\;R}u_{J}\left(y|\theta ,\gamma_{0}\right)u_{J}^{\prime }\left(y|\theta ,\gamma_{0}\right)s_{J}\left(y|\theta ,\gamma_{0}\right)g_{B}^{\left(i\right)\frac{1}{2}}\left(y\right)dy\\ & \equiv & D_{1B}\left(\theta \left(\gamma_{0}\right)\right)+D_{2B}\left(\theta \left(\gamma_{0}\right)\right), \end{array}$$

and $\nabla HD_{B}^{2}\left(\cdot \right)$ is given by

$$\begin{array}{c} \nabla HD_{B}^{2}\left(\theta \left(\gamma_{0}\right)\right)=-\frac{1}{2}\int_{I\;R}u_{J}\left(y|\theta ,\gamma_{0}\right)s_{J}\left(y|\theta ,\gamma_{0}\right)\left(h_{J}^{\frac{1}{2}}\left(y|\theta^{*},\gamma_{0}\right)-g_{B}^{\left(i\right)\frac{1}{2}}\left(y\right)\right)dy. \end{array}$$

Thus,

$$\begin{array}{c} {\left({\hat{\theta }}_{i,B}\left(\gamma_{0}\right)-\theta^{*}\left(\gamma_{0}\right)\right)}^{\prime }=D_{B}^{-1}\left({\tilde{\theta }}_{i,B}\left(\gamma_{0}\right)\right)\nabla HD_{B}^{2}\left(\theta^{*}\left(\gamma_{0}\right)\right). \end{array}$$

By using the identity, $b^{\frac{1}{2}}-a^{\frac{1}{2}}={\left(2a^{\frac{1}{2}}\right)}^{-1}(\left(b-a\right)-{\left(b^{\frac{1}{2}}-a^{\frac{1}{2}}\right)}^{2})$, $\nabla HD_{B}^{2}\left(\theta^{*}\left(\gamma_{0}\right)\right)$ can be expressed as the difference of $T_{B}\left(\gamma_{0}\right)$ and $R_{B}\left(\gamma_{0}\right)$, where

$$\begin{array}{c} T_{B}\left(\gamma_{0}\right)\equiv \frac{1}{4}\int_{I\;R}u_{J}\left(y|\theta^{*},\gamma_{0}\right)\left(h_{J}\left(y|\theta^{*},\gamma_{0}\right)-g_{B}^{\left(i\right)}\left(y\right)\right)dy, \end{array}$$

and

$$\begin{array}{c} R_{B}\left(\gamma_{0}\right)\equiv \frac{1}{4}\int_{I\;R}u_{J}\left(y|\theta^{*},\gamma_{0}\right){\left(h_{J}^{\frac{1}{2}}\left(y|\theta^{*},\gamma_{0}\right)-g_{B}^{\left(i\right)\frac{1}{2}}\left(y\right)\right)}^{2}dy. \end{array}$$

Hence,

$$\begin{array}{c} B^{\frac{1}{2}}{\left({\hat{\theta }}_{i,B}\left(\gamma_{0}\right)-\theta^{*}\left(\gamma_{0}\right)\right)}^{\prime }=A_{1B}\left(\gamma_{0}\right)+A_{2B}\left(\gamma_{0}\right), \end{array}$$

where $A_{1B}\left(\gamma_{0}\right)$ and $A_{2B}\left(\gamma_{0}\right)$ are given in (9). □

**Remark** **2.** *In the rest of the manuscript, we will refer to $A_{2B}\left(\gamma_{0}\right)$ as the remainder term in the representation formula.*

We now turn to the first main result of the manuscript, namely a central limit theorem for ${\hat{\theta }}_{i,B}\left(\gamma_{0}\right)$ as first B→∞ and then $\gamma_{0}\to 0$. As a first step, we note that the Fisher information of the density $h^{*J}\left(\cdot |\theta \right)$ is given by

(15) $$\begin{array}{c} I\left(\theta \right)=\int_{I\;R}v_{J}\left(y|\theta \right)v_{J}^{\prime }\left(y|\theta \right)h^{*J}\left(y|\theta \right)dy. \end{array}$$

Next we state the assumptions needed in the proof of Theorem 1. We separate these conditions as (i) model assumptions, (ii) kernel assumptions, (iii) regularity conditions, (iV) conditions that allow comparison of original data and compressed data.

**Model assumptions on**$h^{*J}\left(\cdot |\theta \right)$

(**D1’**) $h^{*J}\left(\cdot |\theta \right)$ is twice continuously differentiable in θ.

(**D2’**) Assume further that $||\nabla t_{J}\left(\cdot |\theta \right){\left|\right|}_{2}$ is continuous and bounded.

**Kernel assumptions**

(**B1**) K(·) is symmetric about 0 on a compact support and bounded in $L_{2}.$ We denote the support of K(·) by Supp(K).

(**B2**) The bandwidth $c_{B}$ satisfies $c_{B}\to 0$, $B^{\frac{1}{2}}c_{B}\to \infty ,B^{\frac{1}{2}}c_{B}^{2}\to 0$.

**Regularity conditions**

(**M1**) The function $u_{J}\left(\cdot |\theta ,\gamma_{0}\right)s_{J}\left(\cdot |\theta ,\gamma_{0}\right)$ is continuously differentiable and bounded in $L_{2}$ at $\theta^{*}$.

(**M2**) The function ${\dot{u}}_{J}\left(\cdot |\theta ,\gamma_{0}\right)s_{J}\left(\cdot |\theta ,\gamma_{0}\right)$ is continuous and bounded in $L_{2}$ at $\theta^{*}$. In addition, assume that

$$\begin{array}{c} \lim_{B\to \infty }\int_{I\;R}{\left({\dot{u}}_{J}\left(y|\theta_{i,B},\gamma_{0}\right)s_{J}\left(y|\theta_{i,B},\gamma_{0}\right)-{\dot{u}}_{J}\left(y|\theta^{*},\gamma_{0}\right)s_{J}\left(y|\theta^{*},\gamma_{0}\right)\right)}^{2}dy=0. \end{array}$$

(**M3**) The function $u_{J}\left(\cdot |\theta ,\gamma_{0}\right)u_{J}^{\prime }\left(\cdot |\theta ,\gamma_{0}\right)s_{J}\left(\cdot |\theta ,\gamma_{0}\right)$ is continuous and bounded in $L_{2}$ at $\theta^{*}$; also,

$$\begin{array}{c} \lim_{B\to \infty }\int_{I\;R}{\left(u_{J}\left(y|{\hat{\theta }}_{i,B},\gamma_{0}\right)u_{J}^{\prime }\left(y|{\hat{\theta }}_{i,B},\gamma_{0}\right)s_{J}\left(y|\theta_{i,B},\gamma_{0}\right)-u_{J}\left(y|\theta^{*},\gamma_{0}\right)u_{J}^{\prime }\left(y|\theta^{*},\gamma_{0}\right)s_{J}\left(y|\theta^{*},\gamma_{0}\right)\right)}^{2}dy=0. \end{array}$$

(**M4**) Let $\left\{\alpha_{B}:B\geq 1\right\}$ be a sequence diverging to infinity. Assume that

$$\begin{array}{c} \lim_{B\to \infty }B\underset{t\in Supp(K)}{\sup}\;\;P_{\theta^{*}\left(\gamma_{0}\right)}\left(|\Delta -c_{B}t|>\alpha_{B}\right)=0, \end{array}$$

where Supp(K) is the support of the kernel density K(·) and Δ is a generic random variable with density $h_{J}\left(\cdot |\theta^{*},\gamma_{0}\right)$.

(**M5**) Let

$$\begin{array}{c} M_{B}=\underset{\left|y\right|\leq \alpha_{B}}{\sup}\;\underset{t\in Supp(K)}{\sup}\left|\frac{h_{J}\left(y-tc_{B}|\theta^{*},\gamma_{0}\right)}{h_{J}\left(y|\theta^{*},\gamma_{0}\right)}\right|. \end{array}$$

Assume $\underset{B\geq 1}{\sup}M_{B}<\infty$.

(**M6**) The score function has a regular central behavior relative to the smoothing constants, i.e.,

$$\begin{array}{c} \lim_{B\to \infty }{\left(B^{\frac{1}{2}}c_{B}\right)}^{-1}\int_{-\alpha_{B}}^{\alpha_{B}}u_{J}\left(y|\theta^{*},\gamma_{0}\right)dy=0. \end{array}$$

Furthermore,

$$\begin{array}{c} \lim_{B\to \infty }\left(B^{\frac{1}{2}}c_{B}^{4}\right)\int_{-\alpha_{B}}^{\alpha_{B}}u_{J}\left(y|\theta^{*},\gamma_{0}\right)dy=0. \end{array}$$

(**M7**) The density functions are smooth in an $L_{2}$ sense; i.e.,

$$\begin{array}{c} \lim_{B\to \infty }\underset{t\in Supp(K)}{\sup}\int_{I\;R}{\left(u_{J}\left(y+c_{B}t|\theta^{*},\gamma_{0}\right)-u_{J}\left(y|\theta^{*},\gamma_{0}\right)\right)}^{2}h_{J}\left(y|\theta^{*},\gamma_{0}\right)dy=0. \end{array}$$

(**M1’**) The function $v_{J}\left(\cdot |\theta \right)t_{J}\left(\cdot |\theta \right)$ is continuously differentiable and bounded in $L_{2}$ at $\theta^{*}$.

(**M2’**) The function ${\dot{v}}_{J}\left(\cdot |\theta \right)t_{J}\left(\cdot |\theta \right)$ is continuous and bounded in $L_{2}$ at $\theta^{*}$. In addition, assume that

$$\begin{array}{c} \lim_{B\to \infty }\int_{I\;R}{\left({\dot{v}}_{J}\left(y|\theta_{B}\right)t_{J}\left(y|\theta_{B}\right)-{\dot{v}}_{J}\left(y|\theta^{*}\right)t_{J}\left(y|\theta^{*}\right)\right)}^{2}dy=0. \end{array}$$

(**M3’**) The function $v_{J}\left(\cdot |\theta \right)v_{J}^{\prime }\left(\cdot |\theta \right)t_{J}\left(\cdot |\theta \right)$ is continuous and bounded in $L_{2}$ at $\theta^{*}$. also,

$$\begin{array}{c} \lim_{B\to \infty }\int_{I\;R}{\left(v_{J}\left(y|{\hat{\theta }}_{i,B}\right)v_{J}^{\prime }\left(y|{\hat{\theta }}_{i,B}\right)t_{J}\left(y|{\hat{\theta }}_{i,B}\right)-v_{J}\left(y|\theta^{*}\right)v_{J}^{\prime }\left(y|\theta^{*}\right)t_{J}\left(y|\theta^{*}\right)\right)}^{2}dy=0. \end{array}$$

**Assumptions comparing models for original and compressed data**

(**O1**) For all θ∈Θ,

$$\begin{array}{c} \lim_{\gamma_{0}\to 0}\int_{I\;R}{\left(u_{J}\left(y|\theta ,\gamma_{0}\right)u_{J}^{\prime }\left(y|\theta ,\gamma_{0}\right)s_{J}\left(y|\theta ,\gamma_{0}\right)-v_{J}\left(y|\theta \right)v_{J}^{\prime }\left(y|\theta \right)t_{J}\left(y|\theta \right)\right)}^{2}dy=0. \end{array}$$

(**O2**) For all θ∈Θ,

$$\begin{array}{c} \lim_{\gamma_{0}\to 0}\int_{I\;R}{\left({\dot{u}}_{J}\left(y|\theta ,\gamma_{0}\right)s_{J}\left(y|\theta ,\gamma_{0}\right)-{\dot{v}}_{J}\left(y|\theta \right)t_{J}\left(y|\theta \right)\right)}^{2}dy=0. \end{array}$$

**Theorem** **1.** *Assume that the conditions (**B1**)–(**B2**), (**D1**)–(**D2**) , (**D1**’)–(**D2**’), (**M1**)–(**M7**), (**M1’**)–(**M3’**), and (**O1**)–(**O2**) hold. Then, for every 1≤i≤S, the following holds:*

$$\begin{array}{c} \lim_{\gamma_{0}\to 0}\lim_{B\to \infty }P\left(\sqrt{B}({\hat{\theta }}_{i,B}\left(\gamma_{0}\right)-\theta^{*}\left(\gamma_{0}\right))\leq x\right)=P\left(G\leq x\right), \end{array}$$

where G is a bivariate Gaussian random variable with mean 0 and variance $I^{-1}\left(\theta^{*}\right)$, where I(θ) is defined in (15).

Before we embark on the proof of Theorem 1, we first discuss the assumptions. Assumptions (**B1**) and (**B2**) are standard assumptions on the kernel and the bandwidth and are typically employed when investigating the asymptotic behavior of divergence-based estimators (see for instance [1]). Assumptions (**M1**)–(**M7**) and (**M1’**)–(**M3’**) are regularity conditions which are concerned essentially with $L_{2}$ continuity and boundedness of the scores and their derivatives. Assumptions (**O1**)–(**O2**) allow for comparison of $u_{J}\left(\cdot |\theta ,\gamma_{0}\right)$ and $v_{J}\left(\cdot |\theta \right)$. Returning to the proof of Theorem 1, using representation formula, we will first show that $\lim_{\gamma_{0}\to 0}\lim_{B\to \infty }P\left(A_{1B}\left(\gamma_{0}\right)\leq x\right)=P\left(G\leq x\right)$, and then prove that $\lim_{\gamma_{0}\to 0}\lim_{B\to \infty }A_{2B}\left(\gamma_{0}\right)=0$ in probability. We start with the following proposition.

**Proposition** **3.** *Assume that the conditions (**B1**), (**D1**)–(**D2**), (**M1**)–(**M3**), (**M1’**)–(**M3’**), (**M7**) and (**O1**)–(**O2**) hold. Then,*

$$\begin{array}{c} \lim_{\gamma_{0}\to 0}\lim_{B\to \infty }P\left(A_{1B}\left(\gamma_{0}\right)\leq x\right)=P\left(G\leq x\right), \end{array}$$

*where G is given in Theorem 1.*

We divide the proof of Proposition 3 into two lemmas. In the first lemma we will show that

$$\begin{array}{c} \lim_{\gamma_{0}\to 0}\lim_{B\to \infty }D_{B}\left({\tilde{\theta }}_{i,B}\left(\gamma_{0}\right)\right)=\frac{1}{4}I\left(\theta^{*}\right). \end{array}$$

Next in the second lemma we will show that first letting B→∞ and then allowing $\gamma_{0}\to 0,$

$$\begin{array}{c} 4B^{\frac{1}{2}}T_{B}\left(\gamma_{0}\right)\overset{d}{\to }N\left(0,I(\theta^{*})\right). \end{array}$$

We start with the first part.

**Lemma** **2.** *Assume that the conditions (**D1**)–(**D2**), (**D1**’)–(**D2**’), (**M1**)–(**M3**), (**M1’**)–(**M3’**) and (**O1**)–(**O2**) hold. Then, with probability one, the following prevails:*

$$\begin{array}{c} \lim_{\gamma_{0}\to 0}\lim_{B\to \infty }D_{B}\left({\tilde{\theta }}_{i,B}\left(\gamma_{0}\right)\right)=\frac{1}{4}I\left(\theta^{*}\right). \end{array}$$

**Proof.**  Using representation formula in Lemma 1. First fix $\gamma_{0}>0$. It suffices to show

$$\begin{array}{c} \lim_{B\to \infty }D_{1B}\left({\tilde{\theta }}_{i,B}\left(\gamma_{0}\right)\right)=\frac{1}{2}I\left(\theta^{*}\left(\gamma_{0}\right)\right),\;\mathrm{and}\;\lim_{B\to \infty }D_{2B}\left({\tilde{\theta }}_{i,B}\left(\gamma_{0}\right)\right)=-\frac{1}{4}I\left(\theta^{*}\left(\gamma_{0}\right)\right). \end{array}$$

We begin with $D_{1B}\left({\tilde{\theta }}_{i,B}\left(\gamma_{0}\right)\right)$. By algebra, $D_{1B}\left({\tilde{\theta }}_{i,B}\left(\gamma_{0}\right)\right)$ can be expressed as

$$\begin{array}{c} D_{1B}\left({\tilde{\theta }}_{i,B}\left(\gamma_{0}\right)\right)=D_{1B}^{\left(1\right)}\left({\tilde{\theta }}_{i,B}\left(\gamma_{0}\right)\right)+D_{1B}^{\left(2\right)}\left({\tilde{\theta }}_{i,B}\left(\gamma_{0}\right)\right)+D_{1B}^{\left(3\right)}\left(\theta^{*}\left(\gamma_{0}\right)\right),\;\mathrm{where} \end{array}$$

$$\begin{array}{c} D_{1B}^{\left(1\right)}\left({\tilde{\theta }}_{i,B}\left(\gamma_{0}\right)\right)=-\frac{1}{2}\int_{I\;R}{\dot{u}}_{J}\left(y|{\tilde{\theta }}_{i,B},\gamma_{0}\right)s_{J}\left(y|{\tilde{\theta }}_{i,B},\gamma_{0}\right)\left(g_{B}^{\left(i\right)\frac{1}{2}}\left(y\right)-s_{J}\left(y|\theta^{*},\gamma_{0}\right)\right)dy, \end{array}$$

$$\begin{array}{c} D_{1B}^{\left(2\right)}\left({\tilde{\theta }}_{i,B}\left(\gamma_{0}\right)\right)=-\frac{1}{2}\int_{I\;R}({\dot{u}}_{J}\left(y|{\tilde{\theta }}_{i,B},\gamma_{0}\right)s_{J}\left(y|{\tilde{\theta }}_{i,B},\gamma_{0}\right)-{\dot{u}}_{J}\left(y|\theta^{*},\gamma_{0}\right)s_{J}\left(y|\theta^{*},\gamma_{0}\right))h_{J}^{\frac{1}{2}}\left(y|\theta^{*},\gamma_{0}\right)dy, \end{array}$$

$$\begin{array}{c} \mathrm{and}\;D_{1B}^{\left(3\right)}\left(\theta_{B}^{*}\left(\gamma_{0}\right)\right)=-\frac{1}{2}\int_{I\;R}{\dot{u}}_{J}\left(y|\theta^{*},\gamma_{0}\right)h_{J}\left(y|\theta^{*},\gamma_{0}\right)dy=\frac{1}{2}I\left(\theta^{*}\left(\gamma_{0}\right)\right). \end{array}$$

It suffices to show that as B→∞, $D_{1B}^{\left(1\right)}\left({\tilde{\theta }}_{i,B}\left(\gamma_{0}\right)\right)\to 0$, and $D_{1B}^{\left(2\right)}\left({\tilde{\theta }}_{i,B}\left(\gamma_{0}\right)\right)\to 0$. We first consider $D_{1B}^{\left(1\right)}\left({\tilde{\theta }}_{i,B}\left(\gamma_{0}\right)\right)$. By Cauchy-Schwarz inequality and assumption (**M2**), it follows that there exists $0<C_{1}<\infty$,

$$\begin{array}{ccc} \left|D_{1B}^{\left(1\right)}\left({\tilde{\theta }}_{i,B}\left(\gamma_{0}\right)\right)\right| & \leq & \frac{1}{2}{\left\{\int_{I\;R}{\left({\dot{u}}_{J}\left(y|{\tilde{\theta }}_{i,B},\gamma_{0}\right)s_{J}\left(y|{\tilde{\theta }}_{i,B},\gamma_{0}\right)\right)}^{2}dy\right\}}^{\frac{1}{2}}{\left\{\int_{I\;R}{\left(g_{B}^{\left(i\right)\frac{1}{2}}\left(y\right)-s_{J}\left(y|\theta^{*},\gamma_{0}\right)\right)}^{2}dy\right\}}^{\frac{1}{2}}\\ & \leq & C_{1}{\left\{\int_{I\;R}{\left(g_{B}^{\left(i\right)\frac{1}{2}}\left(y\right)-s_{J}\left(y|\theta^{*},\gamma_{0}\right)\right)}^{2}dy\right\}}^{\frac{1}{2}}\to 0, \end{array}$$

where the last convergence follows from the $L_{1}$ convergence of $g_{B}^{\left(i\right)}\left(\cdot \right)$ and $h_{J}\left(\cdot |\theta^{*},\gamma_{0}\right)$. Hence, as B→∞, $D_{1B}^{\left(1\right)}\left({\tilde{\theta }}_{i,B}\left(\gamma_{0}\right)\right)\to 0$. Next we consider $D_{1B}^{\left(2\right)}\left({\tilde{\theta }}_{i,B}\left(\gamma_{0}\right)\right)$. Again, by Cauchy-Schwarz inequality and assumption (**M2**), it follows that $D_{1B}^{\left(2\right)}\left({\tilde{\theta }}_{i,B}\left(\gamma_{0}\right)\right)\to 0$. Hence $D_{1B}\left({\tilde{\theta }}_{i,B}\left(\gamma_{0}\right)\right)\to \frac{1}{2}I\left(\theta^{*}\left(\gamma_{0}\right)\right)$. Turning to $D_{2B}\left({\tilde{\theta }}_{i,B}\left(\gamma_{0}\right)\right)$, by similar argument, using Cauchy-Schwarz inequality and assumption (**M3**), it follows that $D_{2B}\left({\tilde{\theta }}_{i,B}\left(\gamma_{0}\right)\right)\to -\frac{1}{4}I\left(\theta^{*}\left(\gamma_{0}\right)\right)$. Thus, to complete the proof, it is enough to show that

(16) $$\begin{array}{c} \lim_{\gamma_{0}\to 0}\lim_{B\to \infty }D_{1B}\left({\tilde{\theta }}_{i,B}\left(\gamma_{0}\right)\right)=\frac{1}{2}I\left(\theta^{*}\right)\;\mathrm{and}\;\lim_{\gamma_{0}\to 0}\lim_{B\to \infty }D_{2B}\left({\tilde{\theta }}_{i,B}\left(\gamma_{0}\right)\right)=-\frac{1}{4}I\left(\theta^{*}\right). \end{array}$$

We start with the first term of (16). Let

$$\begin{array}{c} J_{1}\left(\gamma_{0}\right)=\int_{I\;R}{\dot{u}}_{J}\left(y|\theta^{*},\gamma_{0}\right)h_{J}\left(y|\theta^{*},\gamma_{0}\right)dy-\int_{I\;R}{\dot{v}}_{J}\left(y|\theta^{*}\right)h^{*J}\left(y|\theta^{*}\right)dy. \end{array}$$

We will show that $\lim_{\gamma_{0}\to 0}J_{1}\left(\gamma_{0}\right)=0$. By algebra, the difference of the above two terms can be expressed as the sum of $J_{11}\left(\gamma_{0}\right)$ and $J_{12}\left(\gamma_{0}\right)$, where

$$\begin{array}{c} J_{11}\left(\gamma_{0}\right)=\int_{I\;R}\left({\dot{u}}_{J}\left(y|\theta^{*},\gamma_{0}\right)s_{J}\left(y|\theta^{*},\gamma_{0}\right)-{\dot{v}}_{J}\left(y|\theta^{*}\right)t_{J}\left(y|\theta^{*}\right)\right)s_{J}\left(y|\theta^{*},\gamma_{0}\right)dy,\;\mathrm{and} \end{array}$$

$$\begin{array}{c} J_{12}\left(\gamma_{0}\right)=\int_{I\;R}{\dot{v}}_{J}\left(y|\theta^{*}\right)t_{J}\left(y|\theta^{*}\right)\left(s_{J}\left(y|\theta^{*},\gamma_{0}\right)-t_{J}\left(y|\theta^{*}\right)\right)dy. \end{array}$$

$J_{11}\left(\gamma_{0}\right)$ converges to zero by Cauchy-Schwarz inequality and assumption (**O2**), and $J_{12}\left(\gamma_{0}\right)$ converges to zero by Cauchy-Schwarz inequality, assumption (**M2’**) and Scheffe’s theorem. Next we consider the second term of (16). Let

$$\begin{array}{c} J_{2}\left(\gamma_{0}\right)=\int_{I\;R}u_{J}\left(y|\theta^{*},\gamma_{0}\right)u_{J}^{\prime }\left(y|\theta^{*},\gamma_{0}\right)h_{J}\left(y|\theta^{*},\gamma_{0}\right)dy-\int_{I\;R}v_{J}\left(y|\theta^{*}\right)v_{J}^{\prime }\left(y|\theta^{*}\right)h^{*J}\left(y|\theta^{*}\right)dy. \end{array}$$

We will show that $\lim_{\gamma_{0}\to 0}J_{2}\left(\gamma_{0}\right)=0$. By algebra, the difference of the above two terms can be expressed as the sum of $J_{21}\left(\gamma_{0}\right)$ and $J_{22}\left(\gamma_{0}\right)$, where

$$\begin{array}{c} J_{21}\left(\gamma_{0}\right)=\int_{I\;R}\left(u_{J}\left(y|\theta^{*},\gamma_{0}\right)u_{J}^{\prime }\left(y|\theta^{*},\gamma_{0}\right)s_{J}\left(y|\theta^{*},\gamma_{0}\right)-v_{J}\left(y|\theta^{*}\right)v_{J}^{\prime }\left(y|\theta^{*}\right)t_{J}\left(y|\theta^{*}\right)\right)s_{J}\left(y|\theta^{*},\gamma_{0}\right)dy, \end{array}$$

$$\begin{array}{c} \mathrm{and}\;J_{22}\left(\gamma_{0}\right)=\int_{I\;R}v_{J}\left(y|\theta^{*}\right)v_{J}^{\prime }\left(y|\theta^{*}\right)t_{J}\left(y|\theta^{*}\right)\left(s_{J}\left(y|\theta^{*},\gamma_{0}\right)-t_{J}\left(y|\theta^{*}\right)\right)dy. \end{array}$$

$J_{11}\left(\gamma_{0}\right)$ converges to zero by Cauchy-Schwarz inequality and assumption (**O1**), and $J_{12}\left(\gamma_{0}\right)$ converges to zero by Cauchy-Schwarz inequality, assumption (**M3’**) and Scheffe’s theorem. Therefore the lemma holds. □

**Lemma** **3.** *Assume that the conditions (**B1**), (**D1**)–(**D2**), (**D1**’)–(**D2**’), (**M1**)–(**M3**), (**M3’**), (**M7**) and (**O1**)–(**O2**) hold. Then, first letting B→∞, and then $\gamma_{0}\to 0,$*

$$\begin{array}{c} 4B^{\frac{1}{2}}T_{B}\left(\gamma_{0}\right)\overset{d}{\to }N\left(0,I(\theta^{*})\right). \end{array}$$

**Proof.**  First fix $\gamma_{0}>0$. Please note that using $\int_{I\;R}u_{J}\left(y|\theta^{*},\gamma_{0}\right)h_{J}\left(y|\theta^{*},\gamma_{0}\right)dy=0$, we have that

$$\begin{array}{ccc} 4B^{\frac{1}{2}}T_{B}\left(\gamma_{0}\right) & = & B^{\frac{1}{2}}\int_{I\;R}u_{J}\left(y|\theta^{*},\gamma_{0}\right)g_{B}^{\left(i\right)}\left(y\right)dy\\ & = & B^{\frac{1}{2}}\int_{I\;R}u_{J}\left(y|\theta^{*},\gamma_{0}\right)\frac{1}{B}\sum_{l=1}^{B}\frac{1}{c_{B}}K\left(\frac{y-Y_{il}}{c_{B}}\right)dy\\ & = & B^{\frac{1}{2}}\frac{1}{B}\sum_{l=1}^{B}\int_{I\;R}u_{J}\left(Y_{il}+c_{B}t|\theta^{*},\gamma_{0}\right)K\left(t\right)dt. \end{array}$$

Therefore,

$$\begin{array}{c} 4B^{\frac{1}{2}}T_{B}\left(\gamma_{0}\right)-B^{\frac{1}{2}}\frac{1}{B}\sum_{l=1}^{B}u_{J}\left(Y_{il}|\theta^{*},\gamma_{0}\right)=B^{\frac{1}{2}}\frac{1}{B}\sum_{l=1}^{B}\int_{I\;R}\left(u_{J}\left(Y_{il}+c_{B}t|\theta^{*},\gamma_{0}\right)-u_{J}\left(Y_{il}|\theta^{*},\gamma_{0}\right)\right)K\left(t\right)dt. \end{array}$$

Since $Y_{il}$’s are i.i.d. across *l*, using Cauchy-Schwarz inequality and assumption (**B1**), we can show that there exists 0<C<∞,

$$\begin{array}{ccc} E{\left[4B^{\frac{1}{2}}T_{B}-B^{\frac{1}{2}}\frac{1}{B}\sum_{l=1}^{B}u_{J}\left(Y_{il}|\theta^{*},\gamma_{0}\right)\right]}^{2} & = & E{\left[\int_{I\;R}\left(u_{J}\left(Y_{i1}+c_{B}t|\theta^{*},\gamma_{0}\right)-u_{J}\left(Y_{i1}|\theta^{*},\gamma_{0}\right)\right)K\left(t\right)dt\right]}^{2}\\ & \leq & CE{\left[{\left\{\int_{I\;R}{\left(u_{J}\left(Y_{i1}+c_{B}t|\theta^{*},\gamma_{0}\right)-u_{J}\left(Y_{i1}|\theta^{*},\gamma_{0}\right)\right)}^{2}dt\right\}}^{\frac{1}{2}}\right]}^{2}\\ & \leq & CE\left[\int_{I\;R}{\left(u_{J}\left(Y_{i1}+c_{B}t|\theta^{*},\gamma_{0}\right)-u_{J}\left(Y_{i1}|\theta^{*},\gamma_{0}\right)\right)}^{2}dt\right]\\ & = & C\int_{I\;R}\int_{I\;R}{\left(u_{J}\left(y+c_{B}t|\theta^{*},\gamma_{0}\right)-u_{J}\left(y|\theta^{*},\gamma_{0}\right)\right)}^{2}h_{J}\left(y|\theta^{*},\gamma_{0}\right)dydt, \end{array}$$

converging to zero as B→∞ by assumption (**M7**). Also, the limiting distribution of $4B^{\frac{1}{2}}T_{B}\left(\gamma_{0}\right)$ is $N(0,I\left(\theta^{*}\left(\gamma_{0}\right)\right))$ as B→∞. Now let $\gamma_{0}\to 0.$ It is enough to show that as $\gamma_{0}\to 0$ the density of $N(0,I\left(\theta^{*}\left(\gamma_{0}\right)\right))$ converges to the density of $N(0,I\left(\theta^{*}\right))$. To this end, it suffices to show that $\lim_{\gamma_{0}\to 0}I\left(\theta^{*}\left(\gamma_{0}\right)\right)=I\left(\theta^{*}\right)$. However, this is established in Lemma 2. Combining the results, the lemma follows. □

**Proof** **of Proposition 3.** The proof of Proposition 3 follows immediately by combining Lemmas 2 and 3. □

We now turn to establishing that the remainder term in the representation formula converges to zero.

**Lemma** **4.** *Assume that the assumptions (**B1**)–(**B2**), (**M1**)–(**M6**) hold. Then*

$$\begin{array}{c} \lim_{\gamma_{0}\to 0}\lim_{B\to \infty }A_{2B}\left(\gamma_{0}\right)=0\;\mathrm{in}\;\mathrm{probability}. \end{array}$$

**Proof.**  Using Lemma 2, it is sufficient to show that $B^{\frac{1}{2}}R_{B}$ converges to 0 in probability as B→∞. Let

$$\begin{array}{c} d_{J}\left(y|\theta^{*}\left(\gamma_{0}\right)\right)=g_{B}^{\left(i\right)\frac{1}{2}}\left(y\right)-s_{J}\left(y|\theta^{*},\gamma_{0}\right). \end{array}$$

Please note that

$$\begin{array}{c} d_{J}^{2}\left(y|\theta^{*}\left(\gamma_{0}\right)\right)\leq 2\left\{{\left(h_{J}\left(y|\theta^{*},\gamma_{0}\right)-E\left[g_{B}^{\left(i\right)}\left(y\right)\right]\right)}^{2}+{\left(E\left[g_{B}^{\left(i\right)}\left(y\right)\right]-g_{B}^{\left(i\right)}\left(y\right)\right)}^{2}\right\}h_{J}^{-1}\left(y|\theta^{*},\gamma_{0}\right). \end{array}$$

Then

$$\begin{array}{ccc} |R_{B}\left(\gamma_{0}\right)|\; & \leq & \;\frac{1}{2}\int_{I\;R}\left|u_{J}\left(y|\theta^{*},\gamma_{0}\right)\right|d_{J}^{2}\left(y|\theta^{*}\left(\gamma_{0}\right)\right)dy\\ & \leq & \frac{1}{2}\int_{-\alpha_{B}}^{\alpha_{B}}|u_{J}\left(y|\theta^{*},\gamma_{0}\right)|d_{J}^{2}\left(y|\theta^{*}\left(\gamma_{0}\right)\right)dy+\frac{1}{2}\int_{\left|y\right|\geq \alpha_{B}}\left|u_{J}\left(y|\theta^{*},\gamma_{0}\right)\right|d_{J}^{2}\left(y|\theta^{*}\left(\gamma_{0}\right)\right)dy\\ & \equiv & R_{1B}\left(\gamma_{0}\right)+R_{2B}\left(\gamma_{0}\right). \end{array}$$

We first deal with $R_{1B}\left(\gamma_{0}\right)$, which can be expressed as the sum of $R_{1B}\left(\gamma_{0}\right)$ and $R_{2B}\left(\gamma_{0}\right)$, where

(17) $$\begin{array}{c} R_{1B}^{\left(1\right)}\left(\gamma_{0}\right)=\int_{-\alpha_{B}}^{\alpha_{B}}\left|u_{J}\left(y|\theta^{*},\gamma_{0}\right)\right|{\left(h_{J}\left(y|\theta^{*},\gamma_{0}\right)-E\left[g_{B}^{\left(i\right)}\left(y\right)\right]\right)}^{2}h_{J}^{-1}\left(y|\theta^{*},\gamma_{0}\right)dy, \end{array}$$

$$\begin{array}{c} \mathrm{and}\;R_{1B}^{\left(2\right)}\left(\gamma_{0}\right)=\int_{-\alpha_{B}}^{\alpha_{B}}\left|u\left(y|\theta^{*},\gamma_{0}\right)\right|{\left(E\left[g_{B}^{\left(i\right)}\left(y\right)\right]-g_{B}^{\left(i\right)}\left(y\right)\right)}^{2}h_{J}^{-1}\left(y|\theta^{*},\gamma_{0}\right)dy. \end{array}$$

Now consider $R_{1B}^{\left(2\right)}$. Let ϵ>0 be arbitrary but fixed. Then, by Markov’s inequality,

(18) $$\begin{array}{ccc} P\left(B^{\frac{1}{2}}R_{1B}^{\left(2\right)}>\epsilon \right) & \leq & \epsilon^{-1}B^{\frac{1}{2}}E\left[R_{1B}^{\left(2\right)}\right]\\ & \leq & \epsilon^{-1}B^{\frac{1}{2}}\int_{\alpha_{B}}^{\alpha_{B}}\left|u_{J}\left(y|\theta^{*},\gamma_{0}\right)\right|\left(\mathrm{Var}\left[g_{B}^{\left(i\right)}\left(y\right)\right]\right)h_{J}^{-1}\left(y|\theta^{*},\gamma_{0}\right)dy. \end{array}$$

Now since $Y_{il}^{\prime }s$ are independent and identically distributed across *l*, it follows that

(19) $$\begin{array}{ccc} \mathrm{Var}\left[g_{B}^{\left(i\right)}\left(y\right)\right] & \leq & \frac{1}{Bc_{B}}\int_{I\;R}K^{2}\left(t\right)h_{J}\left(y-tc_{B}|\theta^{*},\gamma_{0}\right)dt. \end{array}$$

Now plugging (19) into (18), interchanging the order of integration (using Tonelli’s Theorem), we get

$$\begin{array}{c} P\left(B^{\frac{1}{2}}R_{1B}^{\left(2\right)}>\epsilon \right)\leq C{\left(B^{\frac{1}{2}}c_{B}\right)}^{-1}\int_{-\alpha_{B}}^{\alpha_{B}}\left|u_{J}\left(y|\theta^{*},\gamma_{0}\right)\right|dy\to 0, \end{array}$$

where *C* is a universal constant, and the last convergence follows from conditions (**M5**)–(**M6**). We now deal with $R_{1B}^{\left(1\right)}$. To this end, we need to calculate ${\left(E\left[g_{B}^{\left(i\right)}\left(y\right)\right]-h_{J}\left(y|\theta^{*},\gamma_{0}\right)\right)}^{2}$. Using change of variables, two-step Taylor approximation, and assumption (**B1**), we get

(20) $$\begin{array}{ccc} E\left[g_{B}^{\left(i\right)}\left(y\right)\right]-h_{J}\left(y|\theta^{*},\gamma_{0}\right) & = & \int_{I\;R}K\left(t\right)\left(h_{J}\left(y-tc_{B}|\theta^{*},\gamma_{0}\right)-h_{J}\left(y|\theta^{*},\gamma_{0}\right)\right)dt\\ & = & \int_{I\;R}K\left(t\right)\frac{{\left(tc_{B}\right)}^{2}}{2}h_{J}^{\prime \prime }\left(y_{B}^{*}\left(t\right)|\theta^{*},\gamma_{0}\right)dt. \end{array}$$

Now plugging in (20) into (17) and using conditions (**M3**) and (**M6**), we get

(21) $$\begin{array}{c} B^{\frac{1}{2}}R_{1B}^{\left(1\right)}\left(\gamma_{0}\right)\leq CB^{\frac{1}{2}}c_{B}^{4}\int_{-\alpha_{B}}^{\alpha_{B}}\left|u_{J}\left(y|\theta^{*},\gamma_{0}\right)\right|dy. \end{array}$$

Convergence of (21) to 0 now follows from condition (**M6**). We next deal with $R_{2B}\left(\gamma_{0}\right)$. To this end, by writing our the square term of $d_{J}\left(\cdot |\theta^{*}\left(\gamma_{0}\right)\right)$, we have

(22) $$\begin{array}{c} B^{\frac{1}{2}}R_{2B}\left(\gamma_{0}\right)=\int_{\left|y\right|\geq \alpha_{B}}\left|u_{J}\left(y|\theta^{*},\gamma_{0}\right)\right|\left(h_{J}\left(y|\theta^{*},\gamma_{0}\right)+g_{B}^{\left(i\right)}\left(y\right)-s_{J}\left(y|\theta^{*},\gamma_{0}\right)g_{B}^{\left(i\right)\frac{1}{2}}\left(y\right)\right)dy. \end{array}$$

We will show that the RHS of (22) converges to 0 as B→∞. We begin with the first term. Please note that by Cauchy-Schwarz inequality,

$$\begin{array}{ccc} B{\left(\int_{\left|y\right|\geq \alpha_{B}}\left|u_{J}\left(y|\theta^{*},\gamma_{0}\right)\right|h_{J}\left(y|\theta^{*},\gamma_{0}\right)dy\right)}^{2} & \leq & \left\{\int_{I\;R}u_{J}\left(y|\theta^{*},\gamma_{0}\right)u_{J}^{\prime }\left(y|\theta^{*},\gamma_{0}\right)h_{J}\left(y|\theta^{*},\gamma_{0}\right)dy\right\}\\ & \; & \{BP_{\theta^{*}\left(\gamma_{0}\right)}\left(|\Delta \right|\geq \alpha_{B})\}, \end{array}$$

the last term converges to 0 by (**M4**). As for the second term, note that, a.s., by Cauchy-Schwarz inequality,

$$\begin{array}{c} {\left(\int_{\left|y\right|\geq \alpha_{B}}\left|u_{J}\left(y|\theta^{*},\gamma_{0}\right)\right|g_{B}^{\left(i\right)}\left(y\right)dy\right)}^{2}\leq \int_{\left|y\right|\geq \alpha_{B}}u_{J}\left(y|\theta^{*},\gamma_{0}\right)u_{J}^{\prime }\left(y|\theta^{*},\gamma_{0}\right)g_{B}^{\left(i\right)}\left(y\right)dy. \end{array}$$

Now taking the expectation and using Cauchy-Schwarz inequality, one can show that

$$\begin{array}{c} BE{\left[\int_{\left|y\right|\geq \alpha_{m}}\left|u_{J}\left(y|\theta^{*},\gamma_{0}\right)\right|g_{B}^{\left(i\right)}\left(y\right)dy\right]}^{2}\leq a_{B}\int_{I\;R}K\left(t\right)\int_{I\;R}u_{J}\left(y|\theta^{*},\gamma_{0}\right)u_{J}^{\prime }\left(y|\theta^{*},\gamma_{0}\right)h_{J}\left(y-c_{B}t|\theta^{*},\gamma_{0}\right)dydt, \end{array}$$

where $a_{B}=B\underset{z\in Supp(K)}{\sup}P_{\theta^{*}}\left(|\Delta -c_{B}z|>\alpha_{B}\right)$. The convergence to 0 of the RHS of above inequality now follows from condition (**M4**). Finally, by another application of the Cauchy-Schwarz inequality,

$$\begin{array}{c} BE\left[\int_{\left|y\right|\geq \alpha_{m}}\left|u_{J}\left(y|\theta^{*},\gamma_{0}\right)\right|g_{B}^{\left(i\right)\frac{1}{2}}\left(y\right)s_{J}\left(y|\theta^{*},\gamma_{0}\right)dy\right]\leq a_{B}\int_{I\;R}u_{J}\left(y-c_{B}t|\theta^{*},\gamma_{0}\right)u_{J}^{\prime }\left(y-c_{B}t|\theta^{*},\gamma_{0}\right)h_{J}\left(y|\theta^{*},\gamma_{0}\right)dy. \end{array}$$

The convergence of RHS of above inequality to zero follows from (**M4**). Now the lemma follows. □

**Proof** **of Theorem 1.** Recall that

$$\begin{array}{c} B^{\frac{1}{2}}{\left({\hat{\theta }}_{i,B}\left(\gamma_{0}\right)-\theta^{*}\left(\gamma_{0}\right)\right)}^{\prime }=A_{1B}\left(\gamma_{0}\right)+A_{2B}\left(\gamma_{0}\right), \end{array}$$

where $A_{1B}\left(\gamma_{0}\right)$ and $A_{2B}\left(\gamma_{0}\right)$ are given in (9). Proposition 3 shows that $\lim_{\gamma_{0}\to 0}\lim_{B\to \infty }A_{1B}\left(\gamma_{0}\right)=N\left(0,I^{-1}\left(\theta^{*}\right)\right)$; while Lemma 4 shows that $\lim_{\gamma_{0}\to 0}\lim_{B\to \infty }A_{2B}\left(\gamma_{0}\right)=0$ in probability. The result follows from Slutsky’s theorem. □

We next show that by interchanging the limits, namely first allowing $\gamma_{0}$ to converge to 0 and then letting B→∞ the limit distribution of ${\hat{\theta }}_{i,B}\left(\gamma_{0}\right)$ is Gaussian with the same covariance matrix as Theorem 1. We begin with additional assumptions required in the proof of the theorem.

**Regularity conditions**

(**M4’**) Let $\left\{\alpha_{B}:B\geq 1\right\}$ be a sequence diverging to infinity. Assume that

$$\begin{array}{c} \lim_{B\to \infty }B\underset{t\in Supp(K)}{\sup}\;\;P_{\theta^{*}}\left(|\Delta -c_{B}t|>\alpha_{B}\right)=0, \end{array}$$

where Supp(K) is the support of the kernel density K(·) and Δ is a generic random variable with density $h^{*J}\left(\cdot |\theta^{*}\right)$.

(**M5’**) Let

$$\begin{array}{c} M_{B}=\underset{\left|y\right|\leq \alpha_{B}}{\sup}\;\underset{t\in Supp(K)}{\sup}\left|\frac{h^{*J}\left(y-tc_{B}|\theta^{*}\right)}{h^{*J}\left(y|\theta^{*}\right)}\right|. \end{array}$$

Assume that $\underset{B\geq 1}{\sup}M_{B}<\infty$.

(**M6’**) The score function has a regular central behavior relative to the smoothing constants, i.e.,

$$\begin{array}{c} \lim_{B\to \infty }{\left(B^{\frac{1}{2}}c_{B}\right)}^{-1}\int_{-\alpha_{B}}^{\alpha_{B}}v_{J}\left(y|\theta^{*}\right)dy=0. \end{array}$$

Furthermore,

$$\begin{array}{c} \lim_{B\to \infty }\left(B^{\frac{1}{2}}c_{B}^{4}\right)\int_{-\alpha_{B}}^{\alpha_{B}}v_{J}\left(y|\theta^{*}\right)dy=0. \end{array}$$

(**M7’**) The density functions are smooth in an $L_{2}$ sense; i.e.,

$$\begin{array}{c} \lim_{B\to \infty }\underset{t\in Supp(K)}{\sup}\int_{I\;R}{\left(v_{J}\left(y+c_{B}t|\theta^{*}\right)-v_{J}\left(y|\theta^{*}\right)\right)}^{2}h^{*J}\left(y|\theta^{*}\right)dy=0. \end{array}$$

**Assumptions comparing models for original and compressed data**

(**V1**) Assume that $\lim_{\gamma_{0}\to 0}\sup_{y}\left|u_{J}\left(y|\theta^{*},\gamma_{0}\right)-v_{J}\left(y|\theta^{*}\right)\right|=0.$

(**V2**) $v_{J}\left(\cdot |\theta \right)$ is $L_{1}$ continuous in the sense that $X_{n}\overset{p}{\to }X$ implies that $E\left[v_{J}\left(X_{n}|\theta \right)-v_{J}\left(X|\theta \right)\right]=0$, where the expectation is with respect to distribution K(·).

(**V3**) Assume that for all θ∈Θ, $\int_{I\;R}\nabla h^{*J}\left(y|\theta \right)dy<\infty$.

(**V4**) Assume that for all θ∈Θ, $\lim_{\gamma_{0}\to 0}\sup_{y}\left|\frac{s_{J}\left(y|\theta ,\gamma_{0}\right)}{t_{J}\left(y|\theta \right)}-1\right|=0$.

**Theorem** **2.** *Assume that the conditions (**B1**)–(**B2**), (**D1**’)–(**D2**’), (**M1’**)–(**M7’**), (**O1**)–(**O2**) and (**V1**)–(**V4**) hold. Then,*

$$\begin{array}{c} \lim_{B\to \infty }\lim_{\gamma_{0}\to 0}P\left(\sqrt{B}({\hat{\theta }}_{i,B}\left(\gamma_{0}\right)-\theta^{*}\left(\gamma_{0}\right))\leq x\right)=P\left(G\leq x\right), \end{array}$$

where G is a bivariate Gaussian random variable with mean 0 and variance $I^{-1}\left(\theta^{*}\right)$.

We notice that in the above Theorem 2 that we use conditions (**V2**)–(**V4**) which are regularity conditions on the scores of the J− fold convolution of f(·) while (V1) facilitates comparison of the scores of the densities of the compressed data and that of the J−fold convolution. As before, we will first establish (a):

$$\begin{array}{c} \lim_{B\to \infty }\lim_{\gamma_{0}\to 0}P\left(A_{1B}\left(\gamma_{0}\right)\leq x\right)=P\left(G\leq x\right), \end{array}$$

and then (b): $\lim_{B\to \infty }\lim_{\gamma_{0}\to 0}A_{2B}\left(\gamma_{0}\right)=0$ in probability. We start with the proof of (a).

**Proposition** **4.** *Assume that the conditions (**B1**)–(**B2**), (**D1**’)–(**D2**’), (**M1’**)–(**M3’**), (**M7’**), (**O1**)–(**O2**), and (**V1**)–(**V2**) hold. Then,*

$$\begin{array}{c} \lim_{B\to \infty }\lim_{\gamma_{0}\to 0}P\left(A_{1B}\left(\gamma_{0}\right)\leq x\right)=P\left(G\leq x\right). \end{array}$$

We divide the proof of Proposition 4 into two lemmas. In the first lemma, we will show that

$$\begin{array}{c} \lim_{B\to \infty }\lim_{\gamma_{0}\to 0}D_{B}\left({\tilde{\theta }}_{i,B}\left(\gamma_{0}\right)\right)=\frac{1}{4}I\left(\theta^{*}\right). \end{array}$$

In the second lemma, we will show that first let $\gamma_{0}\to 0$, then let B→∞,

$$\begin{array}{c} 4B^{\frac{1}{2}}T_{B}\left(\gamma_{0}\right)\overset{d}{\to }N\left(0,I(\theta^{*})\right). \end{array}$$

**Lemma** **5.** *Assume that the conditions (**B1**)–(**B2**), (**D1**’)–(**D2**’), (**M1’**)–(**M3’**), (**O1**)–(**O2**), and (**V1**)–(**V2**) hold. Then,*

(23) $$\begin{array}{c} \lim_{B\to \infty }\lim_{\gamma_{0}\to 0}D_{B}\left({\tilde{\theta }}_{i,B}\left(\gamma_{0}\right)\right)=\frac{1}{4}I\left(\theta^{*}\right). \end{array}$$

**Proof.**  First fix *B*. Recall that

$$\begin{array}{ccc} D_{B}\left(\theta \left(\gamma_{0}\right)\right) & = & -\frac{1}{2}\int_{I\;R}{\dot{u}}_{J}\left(y|\theta ,\gamma_{0}\right)s_{J}\left(y|\theta ,\gamma_{0}\right){\left(g_{B}^{\left(i\right)}\left(y\right)\right)}^{\frac{1}{2}}dy\\ & \; & -\frac{1}{4}\int_{I\;R}u_{J}\left(y|\theta ,\gamma_{0}\right)u_{J}^{\prime }\left(y|\theta ,\gamma_{0}\right)s_{J}\left(y|\theta ,\gamma_{0}\right){\left(g_{B}^{\left(i\right)}\left(y\right)\right)}^{\frac{1}{2}}dy\\ & \equiv & D_{1B}\left(\theta \left(\gamma_{0}\right)\right)+D_{2B}\left(\theta \left(\gamma_{0}\right)\right). \end{array}$$

By algebra, $D_{1B}\left({\tilde{\theta }}_{i,B}\left(\gamma_{0}\right)\right)$ can be expressed as the sum of $H_{1B}^{\left(1\right)}$, $H_{1B}^{\left(2\right)}$, $H_{1B}^{\left(3\right)}$, $H_{1B}^{\left(4\right)}$ and $H_{1B}^{\left(5\right)}$, where

$$\begin{array}{c} H_{1B}^{\left(1\right)}=-\frac{1}{2}\int_{I\;R}[{\dot{u}}_{J}\left(y|{\tilde{\theta }}_{i,B},\gamma_{0}\right)s_{J}\left(y|{\tilde{\theta }}_{i,B},\gamma_{0}\right)-{\dot{v}}_{J}\left(y|{\tilde{\theta }}_{i,B}\right)t_{J}\left(y|{\tilde{\theta }}_{i,B}\right)]g_{B}^{\left(i\right)\frac{1}{2}}\left(y\right)dy, \end{array}$$

$$\begin{array}{c} H_{1B}^{\left(2\right)}=-\frac{1}{2}\int_{I\;R}[{\dot{v}}_{J}\left(y|{\tilde{\theta }}_{i,B}\right)t_{J}\left(y|{\tilde{\theta }}_{i,B}\right)-{\dot{v}}_{J}\left(y|\theta^{*}\right)t_{J}\left(y|\theta^{*}\right)]g_{B}^{\left(i\right)\frac{1}{2}}\left(y\right)dy, \end{array}$$

$$\begin{array}{c} H_{1B}^{\left(3\right)}=-\frac{1}{2}\int_{I\;R}{\dot{v}}_{J}\left(y|\theta^{*}\right)t_{J}\left(y|\theta^{*}\right)\left[g_{B}^{\left(i\right)\frac{1}{2}}\left(y\right)-h_{J}^{\frac{1}{2}}\left(y|\theta^{*},\gamma_{0}\right)\right]dy, \end{array}$$

$$\begin{array}{c} H_{1B}^{\left(4\right)}=-\frac{1}{2}\int_{I\;R}{\dot{v}}_{J}\left(y|\theta^{*}\right)t_{J}\left(y|\theta^{*}\right)\left[s_{J}\left(y|\theta^{*},\gamma_{0}\right)-t_{J}\left(y|\theta^{*}\right)\right]dy,\;\mathrm{and}\;H_{1B}^{\left(5\right)}=\frac{1}{2}I\left(\theta^{*}\right). \end{array}$$

We will show that

(24) $$\begin{array}{c} \lim_{\gamma_{0}\to 0}D_{1B}\left({\tilde{\theta }}_{i,B}\left(\gamma_{0}\right)\right)=H_{1B}^{\left(2\right)}+\lim_{\gamma_{0}\to 0}H_{1B}^{\left(3\right)}+H_{1B}^{\left(5\right)}, \end{array}$$

where

(25) $$\lim_{\gamma_{0}\to 0}H_{1B}^{\left(3\right)}=-\frac{1}{2}\int_{I\;R}{\dot{v}}_{J}\left(y|\theta^{*}\right)t_{J}\left(y|\theta^{*}\right)\left[g_{B}^{*\frac{1}{2}}\left(y\right)-t_{J}\left(y|\theta^{*}\right)\right]dy\;\mathrm{and}$$

$g_{B}^{*}\left(\cdot \right)$ is given in (7). First consider $H_{1B}^{\left(1\right)}$. it converges to zero as $\gamma_{0}\to 0$ by Cauchy-Schwarz inequality and assumption (**O2**). Next we consider $H_{1B}^{\left(3\right)}$. We will first show that

$$\begin{array}{c} \lim_{\gamma_{0}\to 0}-\frac{1}{2}\int_{I\;R}{\dot{v}}_{J}\left(y|\theta^{*}\right)t_{J}\left(y|\theta^{*}\right)g_{B}^{\left(i\right)\frac{1}{2}}\left(y\right)dy=-\frac{1}{2}\int_{I\;R}{\dot{v}}_{J}\left(y|\theta^{*}\right)t_{J}\left(y|\theta^{*}\right)g_{B}^{*\frac{1}{2}}\left(y\right)dy. \end{array}$$

To this end, notice that by Cauchy-Schwarz inequality and boundedness of ${\dot{v}}_{J}\left(y|\theta^{*}\right)t_{J}\left(y|\theta^{*}\right)$ in $L_{2}$, it follows that there exists a constant *C* such that

$$\begin{array}{ccc} \left|\int_{I\;R}{\dot{v}}_{J}\left(y|\theta^{*}\right)t_{J}\left(y|\theta^{*}\right)\left[g_{B}^{\left(i\right)\frac{1}{2}}\left(y\right)-g_{B}^{*\frac{1}{2}}\left(y\right)\right]dy\right| & \leq & C{\left\{\int_{I\;R}{\left(g_{B}^{\left(i\right)\frac{1}{2}}\left(y\right)-g_{B}^{*\frac{1}{2}}\left(y\right)\right)}^{2}dy\right\}}^{\frac{1}{2}}\\ & \leq & C{\left\{\int_{I\;R}\left|g_{B}^{\left(i\right)}\left(y\right)-g_{B}^{*}\left(y\right)\right|dy\right\}}^{\frac{1}{2}}. \end{array}$$

It suffices to show that $g_{B}^{\left(i\right)}\left(\cdot \right)$ converges to $g_{B}^{*}\left(\cdot \right)$ in $L_{1}$. Since

$$\begin{array}{c} \int_{I\;R}\left|g_{B}^{\left(i\right)}\left(y\right)-g_{B}^{*}\left(y\right)\right|dy=2-2\int_{I\;R}\min\left\{g_{B}^{\left(i\right)}\left(y\right),g_{B}^{*}\left(y\right)\right\}dy, \end{array}$$

and $\min\left\{g_{B}^{\left(i\right)}\left(y\right),g_{B}^{*}\left(y\right)\right\}\leq g_{B}^{*}\left(y\right)$, by dominated convergence theorem, $g_{B}^{\left(i\right)}\left(\cdot \right)\overset{L_{1}}{\to }g_{B}^{*}\left(\cdot \right)$. Next we will show that

$$\begin{array}{c} \lim_{\gamma_{0}\to 0}-\frac{1}{2}\int_{I\;R}{\dot{v}}_{J}\left(y|\theta^{*}\right)t_{J}\left(y|\theta^{*}\right)s_{J}\left(y|\theta^{*},\gamma_{0}\right)dy=-\frac{1}{2}\int_{I\;R}{\dot{v}}_{J}\left(y|\theta^{*}\right)t_{J}\left(y|\theta^{*}\right)t_{J}\left(y|\theta^{*}\right)dy. \end{array}$$

In addition, by Cauchy-Schwarz inequality, boundedness of ${\dot{v}}_{J}\left(y|\theta^{*}\right)t_{J}\left(y|\theta^{*}\right)$ in $L_{2}$ and Scheffe’s theorem, we have that $\int_{I\;R}{\dot{v}}_{J}\left(y|\theta^{*}\right)h_{J}^{\frac{1}{2}}\left(y|\theta^{*},\gamma_{0}\right)\left(s_{J}\left(y\right|\theta^{*}\left|\gamma_{0}\right)-t_{J}\left(y|\theta^{*}\right)\right)dy$ converges to zero as $\gamma_{0}\to 0.$ Next we consider $H_{1B}^{\left(4\right)}$. it converges to zero by Cauchy-Schwarz inequality and assumption (**M2’**). Thus (24) holds. Now let B→∞, we will show that $\lim_{B\to \infty }H_{1B}^{\left(2\right)}=0$ and $\lim_{B\to \infty }\lim_{\gamma_{0}\to 0}H_{1B}^{\left(3\right)}=0.$ First consider $\lim_{B\to \infty }H_{1B}^{\left(2\right)}$. It converges to zero by Cauchy-Schwarz inequality and assumption (**M2’**). Next we consider $\lim_{B\to \infty }\lim_{\gamma_{0}\to 0}H_{1B}^{\left(3\right)}$. It converges to zero by Cauchy-Schwarz inequality and $L_{1}$ convergence of $g_{B}^{*}\left(\cdot \right)$ and $h^{*J}\left(\cdot |\theta^{*}\right)$. Therefore $\lim_{B\to \infty }\lim_{\gamma_{0}\to 0}D_{1B}\left({\tilde{\theta }}_{i,B}\left(\gamma_{0}\right)\right)=\frac{1}{2}I\left(\theta^{*}\right)$.

We now turn to show that $\lim_{B\to \infty }\lim_{\gamma_{0}\to 0}D_{2B}\left({\tilde{\theta }}_{i,B}\left(\gamma_{0}\right)\right)=-\frac{1}{4}I\left(\theta^{*}\right)$. First fix *B* and express $D_{2B}\left({\tilde{\theta }}_{i,B}\left(\gamma_{0}\right)\right)$ as the sum of $H_{2B}^{\left(1\right)}$, $H_{2B}^{\left(2\right)}$, $H_{2B}^{\left(3\right)}$, $H_{2B}^{\left(4\right)}$, and $H_{2B}^{\left(5\right)}$, where

$$\begin{array}{c} H_{2B}^{\left(1\right)}=-\frac{1}{4}\int_{I\;R}\left[u_{J}\left(y|{\tilde{\theta }}_{i,B},\gamma_{0}\right)u_{J}^{\prime }\left(y|{\tilde{\theta }}_{i,B},\gamma_{0}\right)s_{J}\left(y|{\tilde{\theta }}_{i,B},\gamma_{0}\right)-v_{J}\left(y|{\tilde{\theta }}_{i,B}\right)v_{J}^{\prime }\left(y|{\tilde{\theta }}_{i,B}\right)t_{J}\left(y|{\tilde{\theta }}_{i,B}\right)\right]g_{B}^{\left(i\right)\frac{1}{2}}\left(y\right)dy, \end{array}$$

$$\begin{array}{c} H_{2B}^{\left(2\right)}=-\frac{1}{4}\int_{I\;R}\left[v_{J}\left(y|{\tilde{\theta }}_{i,B}\right)v_{J}^{\prime }\left(y|{\tilde{\theta }}_{i,B}\right)t_{J}\left(y|{\tilde{\theta }}_{i,B}\right)-v_{J}\left(y|\theta^{*}\right)v_{J}^{\prime }\left(y|\theta^{*}\right)t_{J}\left(y|\theta^{*}\right)\right]g_{B}^{\left(i\right)\frac{1}{2}}\left(y\right)dy, \end{array}$$

$$\begin{array}{c} H_{2B}^{\left(3\right)}=-\frac{1}{4}\int_{I\;R}v_{J}\left(y|\theta^{*}\right)v_{J}^{\prime }\left(y|\theta^{*}\right)t_{J}\left(y|\theta^{*}\right)\left[g_{B}^{\left(i\right)\frac{1}{2}}\left(y\right)-h_{J}^{\frac{1}{2}}\left(y|\theta^{*},\gamma_{0}\right)\right]dy, \end{array}$$

$$\begin{array}{c} H_{2B}^{\left(4\right)}=-\frac{1}{4}\int_{I\;R}v_{J}\left(y|\theta^{*}\right)v_{J}^{\prime }\left(y|\theta^{*}\right)t_{J}\left(y|\theta^{*}\right)\left[s_{J}\left(y|\theta^{*},\gamma_{0}\right)-t_{J}\left(y|\theta^{*}\right)\right]dy,\;\mathrm{and}\;H_{2B}^{\left(5\right)}=-\frac{1}{4}I\left(\theta^{*}\right). \end{array}$$

We will show that

(26) $$\begin{array}{c} \lim_{\gamma_{0}\to 0}D_{2B}\left({\tilde{\theta }}_{i,B}\left(\gamma_{0}\right)\right)=H_{2B}^{\left(2\right)}+\lim_{\gamma_{0}\to 0}H_{2B}^{\left(3\right)}+H_{2B}^{\left(5\right)},\;\mathrm{where} \end{array}$$

(27) $$\lim_{\gamma_{0}\to 0}H_{2B}^{\left(3\right)}=-\frac{1}{2}\int_{I\;R}v_{J}\left(y|\theta^{*}\right)v_{J}^{\prime }\left(y|\theta^{*}\right)t_{J}\left(y|\theta^{*}\right)\left[g_{B}^{*\frac{1}{2}}\left(y\right)-t_{J}\left(y|\theta^{*}\right)\right]dy.$$

First consider $H_{2B}^{\left(1\right)}$. It converges to zero as $\gamma_{0}\to 0$ by Cauchy-Schwarz inequality and assumption (**O1**). Next consider $H_{2B}^{\left(3\right)}$. By similar argument as above and boundedness of $v_{J}^{2}\left(y|\theta^{*}\right)t_{J}\left(y|\theta^{*}\right)$, it follows that (27) holds. Next consider $H_{2B}^{\left(4\right)}$. It converges to zero as $\gamma_{0}\to 0$ by Cauchy-Schwarz inequality and assumption (**M3’**). Now let B→∞, we will show that $\lim_{B\to \infty }H_{2B}^{\left(2\right)}=0$ and $\lim_{B\to \infty }\lim_{\gamma_{0}\to 0}H_{2B}^{\left(3\right)}=0$. First consider $H_{2B}^{\left(2\right)}$. It converges to zero by Cauchy-Schwarz inequality and assumption (**M3’**) as B→∞. Finally consider $\lim_{B\to \infty }\lim_{\gamma_{0}\to 0}H_{2B}^{\left(3\right)}$. It converges to zero by Cauchy-Schwarz inequality and $L_{1}$ convergence of $g_{B}^{*}\left(\cdot \right)$ and $h^{*J}\left(\cdot |\theta^{*}\right)$. Thus $\lim_{B\to \infty }\lim_{\gamma_{0}\to 0}D_{2B}\left({\tilde{\theta }}_{i,B}\left(\gamma_{0}\right)\right)=-\frac{1}{4}I\left(\theta^{*}\right)$. Now letting B→∞, the proof of (23) follows using arguments similar to the one in Lemma 2. □

**Lemma** **6.** *Assume that the conditions (**B1**)–(**B2**),(**D1**’)–(**D2**’), (**M1’**)–(**M3’**), (**M7’**), (**O1**)–(**O2**), and (**V1**)–(**V2**) hold. Then, first letting B→∞, and then letting $\gamma_{0}\to 0,$*

(28) $$\begin{array}{c} 4B^{\frac{1}{2}}T_{B}\left(\gamma_{0}\right)\overset{d}{\to }N\left(0,I(\theta^{*})\right). \end{array}$$

**Proof.**  First fix *B*. We will show that as $\gamma_{0}\to 0,$

$$\begin{array}{c} 4B^{\frac{1}{2}}T_{B}\left(\gamma_{0}\right)\overset{d}{\to }\int_{I\;R}v_{J}\left(y|\theta^{*}\right)g_{B}^{*}\left(y\right)dy. \end{array}$$

First observe that

(29) $$\begin{array}{ccc} 4B^{\frac{1}{2}}T_{B}\left(\gamma_{0}\right)-\int_{I\;R}v_{J}\left(y|\theta^{*}\right)g_{B}^{*}\left(y\right)dy & = & \int_{I\;R}\left[u_{J}\left(y|\theta^{*},\gamma_{0}\right)-v_{J}\left(y|\theta^{*}\right)\right]g_{B}^{\left(i\right)}\left(y\right)dy \end{array}$$

(30) $$\begin{array}{cc} \; & +\int_{I\;R}v_{J}\left(y|\theta^{*}\right)\left[g_{B}^{\left(i\right)}\left(y\right)-g_{B}^{*}\left(y\right)\right]dy. \end{array}$$

We will show that the RHS of (29) converges to zero as $\gamma_{0}\to 0$ and the RHS of (30) converges to zero in probability as $\gamma_{0}\to 0$. First consider the RHS of (29). Since

$$\begin{array}{ccc} \int_{I\;R}\left[u_{J}\left(y|\theta^{*},\gamma_{0}\right)-v_{J}\left(y|\theta^{*}\right)\right]g_{B}^{\left(i\right)}\left(y\right)dy & \leq & \int_{I\;R}\underset{y}{\sup}\left|u_{J}\left(y|\theta^{*},\gamma_{0}\right)-v_{J}\left(y|\theta^{*}\right)\right|g_{B}^{\left(i\right)}\left(y\right)dy, \end{array}$$

which converges to zero as $\gamma_{0}\to 0$ by assumption (**V1**). Next consider the RHS of (30). Since

$$\begin{array}{ccc} \int_{I\;R}v_{J}\left(y|\theta^{*}\right)\left[g_{B}^{\left(i\right)}\left(y\right)-g_{B}^{*}\left(y\right)\right]dy & = & \frac{1}{B}\sum_{l=1}^{B}\int_{I\;R}\left[v_{J}\left(Y_{il}+uc_{B}\right)-v_{J}\left(Y_{il}^{*}+uc_{B}\right)\right]K\left(u\right)du. \end{array}$$

By assumption (**V2**), it follows that as $\gamma_{0}\to 0$, (30) converges to zero in probability. Now letting B→∞, we have

$$\begin{array}{c} B^{\frac{1}{2}}\int_{I\;R}v_{J}\left(y|\theta^{*}\right)g_{B}^{*}\left(y\right)dy-B^{\frac{1}{2}}\frac{1}{B}\sum_{l=1}^{B}v_{J}\left(Y_{il}^{*}|\theta^{*}\right)=B^{\frac{1}{2}}\frac{1}{B}\sum_{l=1}^{B}\int_{I\;R}\left(v_{J}\left(Y_{il}^{*}+c_{B}t|\theta^{*}\right)-v_{J}\left(Y_{il}^{*}|\theta^{*}\right)\right)K\left(t\right)dt, \end{array}$$

and

$$\begin{array}{ccc} E{\left[B^{\frac{1}{2}}\int_{I\;R}v_{J}\left(y|\theta^{*}\right)g_{B}^{*}\left(y\right)dy-B^{\frac{1}{2}}\frac{1}{B}\sum_{l=1}^{B}v_{J}\left(Y_{il}^{*}|\theta^{*}\right)\right]}^{2} & = & E{\left[B^{\frac{1}{2}}\frac{1}{B}\sum_{l=1}^{B}\int_{I\;R}\left(v_{J}\left(Y_{il}^{*}+c_{B}t|\theta^{*}\right)-v_{J}\left(Y_{il}^{*}|\theta^{*}\right)\right)K\left(t\right)dt\right]}^{2}\\ & \leq & CE\left[\int_{I\;R}{\left(v_{J}\left(Y_{i1}^{*}+c_{B}t|\theta^{*}\right)-v_{J}\left(Y_{i1}^{*}|\theta^{*}\right)\right)}^{2}dt\right]\\ & = & C\int_{I\;R}\int_{I\;R}{\left(v_{J}\left(y+c_{B}t|\theta^{*}\right)-v_{J}\left(y|\theta^{*}\right)\right)}^{2}h^{*J}\left(y|\theta^{*}\right)dydt\\ & \to & 0\;\mathrm{as}\;B\to \infty , \end{array}$$

where the last convergence follows by assumption (**M7’**). Hence, using the Central limit theorem for independent and identically distributed random variables it follows that the limiting distribution of $B^{\frac{1}{2}}\int_{I\;R}v_{J}\left(y|\theta^{*}\right)g_{B}^{*}\left(y\right)dy$ is $N(0,I\left(\theta^{*}\right))$, proving the lemma. □

**Proof** **of** **Proposition** **4.** The proof of Proposition 4 follows by combining Lemmas 5 and 6. □

**Lemma** **7.** *Assume that the conditions (**M1’**)–(**M6’**) and (**V1**)–(**V4**) hold. Then,*

$$\begin{array}{c} \lim_{B\to \infty }\lim_{\gamma_{0}\to 0}A_{2B}\left(\gamma_{0}\right)=0\;\mathrm{in}\;\mathrm{probability}. \end{array}$$

**Proof.**  First fix *B*. Let

$$\begin{array}{c} H_{B}\left(\gamma_{0}\right)=\int_{I\;R}u_{J}\left(y|\theta^{*},\gamma_{0}\right){\left[h_{J}^{\frac{1}{2}}\left(y|\theta^{*},\gamma_{0}\right)-g_{B}^{\left(i\right)}\left(y\right)\right]}^{2}dy-\int_{I\;R}v_{J}\left(y|\theta^{*}\right){\left[t_{J}\left(y|\theta^{*}\right)-g_{B}^{*}\left(y\right)\right]}^{2}dy. \end{array}$$

we will show that as $\gamma_{0}\to 0$, $H_{B}\left(\gamma_{0}\right)\to 0$. By algebra, $H_{B}\left(\gamma_{0}\right)$ can be written as the sum of $H_{1B}\left(\gamma_{0}\right)$ and $H_{2B}\left(\gamma_{0}\right)$, where

$$\begin{array}{c} H_{1B}\left(\gamma_{0}\right)=\int_{I\;R}\left(u_{J}\left(y|\theta^{*},\gamma_{0}\right)-v_{J}\left(y|\theta^{*}\right)\right){\left[h_{J}^{\frac{1}{2}}\left(y|\theta^{*},\gamma_{0}\right)-g_{B}^{\left(i\right)}\left(y\right)\right]}^{2}dy,\;\mathrm{and} \end{array}$$

$$\begin{array}{c} H_{2B}\left(\gamma_{0}\right)=\int_{I\;R}v_{J}\left(y|\theta^{*}\right){\left[h_{J}^{\frac{1}{2}}\left(y|\theta^{*},\gamma_{0}\right)-g_{B}^{\left(i\right)}\left(y\right)\right]}^{2}dy. \end{array}$$

First consider $H_{1B}\left(\gamma_{0}\right)$. It is bounded above by $C\sup_{y}\left|u_{J}\left(y|\theta^{*},\gamma_{0}\right)-v_{J}\left(y|\theta^{*}\right)\right|$, which converges to zero as $\gamma_{0}\to 0$ by assumption (**V1**), where *C* is a constant. Next consider $H_{2B}\left(\gamma_{0}\right)$. We will show that $H_{2B}\left(\gamma_{0}\right)$ converges to

$$\begin{array}{c} \int_{I\;R}v_{J}\left(y|\theta^{*}\right){\left[t_{J}\left(y|\theta^{*},\gamma_{0}\right)-g_{B}^{*}\left(y\right)\right]}^{2}dy. \end{array}$$

In fact, the difference of $H_{2B}\left(\gamma_{0}\right)$ and the above formula can be expressed as the sum of $H_{2B}^{\left(1\right)}\left(\gamma_{0}\right)$, $H_{2B}^{\left(2\right)}\left(\gamma_{0}\right),$ and $H_{2B}^{\left(3\right)}\left(\gamma_{0}\right)$, where

$$\begin{array}{c} H_{2B}^{\left(1\right)}\left(\gamma_{0}\right)=\int_{I\;R}v_{J}\left(y|\theta^{*}\right)\left(h_{J}\left(y|\theta^{*},\gamma_{0}\right)-h^{*J}\left(y|\theta^{*}\right)\right)dy, \end{array}$$

$$\begin{array}{c} H_{2B}^{\left(2\right)}\left(\gamma_{0}\right)=\int_{I\;R}v_{J}\left(y|\theta^{*}\right)\left(g_{B}^{\left(i\right)}\left(y\right)-g_{B}^{*}\left(y\right)\right)dy,\;\mathrm{and} \end{array}$$

$$\begin{array}{c} H_{2B}^{\left(3\right)}\left(\gamma_{0}\right)=\int_{I\;R}v_{J}\left(y|\theta^{*}\right)\left(h_{J}^{\frac{1}{2}}\left(y|\theta^{*},\gamma_{0}\right)g_{B}^{\left(i\right)}\left(y\right)-t_{J}\left(y|\theta^{*},\gamma_{0}\right)g_{B}^{*}\left(y\right)\right)dy. \end{array}$$

First consider $H_{2B}^{\left(1\right)}\left(\gamma_{0}\right)$. Please note that

$$\begin{array}{ccc} \left|H_{2B}^{\left(1\right)}\left(\gamma_{0}\right)\right| & \leq & \int_{I\;R}\left|\nabla h^{*J}\left(y|\theta^{*}\right)\right|\left|\frac{h_{J}\left(y|\theta^{*},\gamma_{0}\right)}{h^{*J}\left(y|\theta^{*}\right)}-1\right|dy\\ & \leq & \left\{{\left(\underset{y}{\sup}\left|\frac{s_{J}\left(y|\theta ,\gamma_{0}\right)}{t_{J}\left(y|\theta \right)}-1\right|\right)}^{2}+2\underset{y}{\sup}\left|\frac{s_{J}\left(y|\theta ,\gamma_{0}\right)}{t_{J}\left(y|\theta \right)}-1\right|\right\}\int_{I\;R}\left|\nabla h^{*J}\left(y|\theta^{*}\right)\right|dy, \end{array}$$

which converges to 0 as $\gamma_{0}\to 0$ by assumptions (**V3**) and (**V4**). Next we consider $H_{2B}^{\left(2\right)}\left(\gamma_{0}\right)$. Since

$$\begin{array}{c} H_{2B}^{\left(2\right)}\left(\gamma_{0}\right)=\frac{1}{B}\sum_{l=1}^{B}\int_{I\;R}\left(v_{J}\left(Y_{il}+uc_{B}|\theta^{*}\right)-v_{J}\left(Y_{il}^{*}+uc_{B}|\theta^{*}\right)\right)K\left(u\right)du, \end{array}$$

which converges to zero as $\gamma_{0}\to 0$ due to assumption (**V2**). Finally consider $H_{2B}^{\left(3\right)}\left(\gamma_{0}\right)$, which can be expressed as the sum of $L_{1B}\left(\gamma_{0}\right)$ and $L_{2B}$, where

$$\begin{array}{c} L_{1B}\left(\gamma_{0}\right)=\int_{I\;R}v_{J}\left(y|\theta^{*}\right)\left(h_{J}^{\frac{1}{2}}\left(y|\theta^{*},\gamma_{0}\right)-t_{J}\left(y|\theta^{*}\right)\right)g_{B}^{\left(i\right)\frac{1}{2}}\left(y\right)dy,\;\mathrm{and} \end{array}$$

$$\begin{array}{c} L_{2B}=\int_{I\;R}v_{J}\left(y|\theta^{*}\right)t_{J}\left(y|\theta^{*}\right)\left(g_{B}^{\left(i\right)\frac{1}{2}}\left(y\right)-g_{B}^{*\frac{1}{2}}\left(y\right)\right)dy. \end{array}$$

First consider $L_{1B}\left(\gamma_{0}\right)$. Notice that

$$\begin{array}{c} \left|L_{1B}\left(\gamma_{0}\right)\right|\leq \underset{y}{\sup}\left|\frac{s_{J}\left(y|\theta ,\gamma_{0}\right)}{t_{J}\left(y|\theta \right)}-1\right|\int_{I\;R}v_{J}\left(y|\theta^{*}\right)t_{J}\left(y|\theta^{*}\right)g_{B}^{\left(i\right)\frac{1}{2}}\left(y\right)dy\to 0, \end{array}$$

where the last convergence follows by Cauchy-Schwarz inequality and assumption (**V4**). Next we consider $L_{2B}$. By Cauchy-Schwarz inequality, it is bounded above by

(31) $$\begin{array}{c} {\left\{\int_{I\;R}v_{J}\left(y|\theta^{*}\right)v_{J}^{\prime }\left(y|\theta^{*}\right)h^{*J}\left(y|\theta^{*}\right)dy\right\}}^{\frac{1}{2}}{\left\{\int_{I\;R}{\left(g_{B}^{\left(i\right)\frac{1}{2}}\left(y\right)-g_{B}^{*\frac{1}{2}}\left(y\right)\right)}^{2}dy\right\}}^{\frac{1}{2}}. \end{array}$$

Equation (31) converges to zero as $\gamma_{0}\to 0$ by boundedness of $\int_{I\;R}v_{J}\left(y|\theta^{*}\right)v_{J}^{\prime }\left(y|\theta^{*}\right)h^{*J}\left(y|\theta^{*}\right)dy$ and $L_{1}$ convergence between $g_{B}^{\left(i\right)}\left(\cdot \right)$ and $g_{B}^{*}\left(\cdot \right)$, where the $L_{1}$ convergence has already been established in Lemma 5. Now letting B→∞, following similar argument as Lemma 4 and assumptions (**M1’**)–(**M6’**), the lemma follows. □

**Proof** **of** **Theorem** **2.** Recall that

$$\begin{array}{c} B^{\frac{1}{2}}{\left({\hat{\theta }}_{i,B}\left(\gamma_{0}\right)-\theta^{*}\left(\gamma_{0}\right)\right)}^{\prime }=A_{1B}\left(\gamma_{0}\right)+A_{2B}\left(\gamma_{0}\right). \end{array}$$

Proposition 4 shows that first letting $\gamma_{0}\to 0,$ then B→∞, $A_{1B}\left(\gamma_{0}\right)\overset{d}{\to }N\left(0,I^{-1}\left(\theta^{*}\right)\right)$; while Lemma 7 shows that $\lim_{B\to \infty }\lim_{\gamma_{0}\to 0}A_{2B}\left(\gamma_{0}\right)=0$ in probability. The theorem follows from Slutsky’s theorem. □

**Remark** **3.** *The above two theorems (Theorems 1 and 2) do not immediately imply the double limit exists. This requires stronger conditions and more delicate calculations and will be considered elsewhere.*

### 3.5. Robustness of MHDE

In this section, we describe the robustness properties of MHDE for compressed data. Accordingly, let $h_{J,\alpha ,z}\left(\cdot |\theta ,\gamma_{0}\right)\equiv \left(1-\alpha \right)h_{J}\left(\cdot |\theta ,\gamma_{0}\right)+\alpha \eta_{z}$, where $\eta_{z}$ denotes the uniform density on the interval (z−ϵ,z+ϵ), where ϵ>0 is small, θ∈Θ, α∈(0,1), and z∈IR. Also, let $s_{J,\alpha ,z}\left(y|\theta ,\gamma_{0}\right)=h_{J,\alpha ,z}^{\frac{1}{2}}\left(y|\theta ,\gamma_{0}\right)$, $u_{J,\alpha ,z}\left(y|\theta ,\gamma_{0}\right)=\nabla \log h_{J,\alpha ,z}\left(y|\theta ,\gamma_{0}\right)$, $h_{\alpha ,z}^{*J}\left(\cdot |\theta \right)\equiv \left(1-\alpha \right)h^{*J}\left(\cdot |\theta \right)+\alpha \eta_{z}$, $s_{\alpha ,z}^{*J}\left(\cdot |\theta \right)=h_{\alpha ,z}^{*J\frac{1}{2}}\left(\cdot |\theta \right)$, and $u_{\alpha ,z}^{*J}=\nabla \log h_{\alpha ,z}^{*J}\left(\cdot |\theta \right)$. Before we state the theorem, we describe certain additional assumptions-which are essentially $L_{2}-$ continuity conditions-that are needed in the proof.

**Model assumptions for robustness analysis**

(**O3**) For α∈[0,1] and all θ∈Θ,

$$\begin{array}{c} \lim_{\gamma_{0}\to 0}\int_{I\;R}{\left({\dot{u}}_{J,\alpha ,z}\left(y|\theta ,\gamma_{0}\right)s_{J,\alpha ,z}\left(y|\theta ,\gamma_{0}\right)-{\dot{u}}_{\alpha ,z}^{*J}\left(y|\theta \right)s_{\alpha ,z}^{*J}\left(y|\theta \right)\right)}^{2}dy=0. \end{array}$$

(**O4**) For α∈[0,1] and all θ∈Θ,

$$\begin{array}{c} \lim_{\gamma_{0}\to 0}\int_{I\;R}{\left(u_{J,\alpha ,z}\left(y|\theta ,\gamma_{0}\right)u_{J,\alpha ,z}^{\prime }\left(y|\theta ,\gamma_{0}\right)s_{J,\alpha ,z}\left(y|\theta ,\gamma_{0}\right)-u_{\alpha ,z}^{*J}\left(y|\theta \right)u_{\alpha ,z}^{*J^{\prime }}\left(y|\theta \right)s_{\alpha ,z}^{*J}\left(y|\theta \right)\right)}^{2}dy=0. \end{array}$$

**Theorem** **3.** *(i) Let α∈(0,1), and assume that for all θ∈Θ, and assume that the assumptions of Proposition 1 hold, also assume that $T(h_{J,\alpha ,z}\left(\cdot |\theta ,\gamma_{0}\right))$ is unique for all z. Then, $T(h_{J,\alpha ,z}\left(\cdot |\theta ,\gamma_{0}\right))$ is a bounded, continuous function of z and*

(32) $$\begin{array}{c} \lim_{\gamma_{0}\to 0}\lim_{|z|\to \infty }T\left(h_{J,\alpha ,z}\left(\cdot |\theta ,\gamma_{0}\right)\right)=\theta ; \end{array}$$

*(ii) Assume further that the conditions (**V1**), (**M2**)-(**M3**), and (**O3**)-(**O4**) hold. Then,*

$$\begin{array}{c} \lim_{\gamma_{0}\to 0}\lim_{\alpha \to 0}\alpha^{-1}\left[T(h_{J,\alpha ,z}\left(\cdot |\theta ,\gamma_{0}\right))-\theta \right]={\left[I(\theta )\right]}^{-1}\int_{I\;R}\left[\eta_{z}\left(y\right)v_{J}\left(y|\theta \right)\right]dy, \end{array}$$

**Proof.**  Let $\theta_{z}\left(\gamma_{0}\right)$ denote $T(h_{J,\alpha ,z}\left(\cdot |\theta ,\gamma_{0}\right))$ and let $\theta_{z}$ denote $T(h_{\alpha ,z}^{*J}\left(\cdot |\theta \right))$ We first show that (32) holds. Let $\gamma_{0}\geq 0$ be fixed. Then, by triangle inequality,

(33) $$\begin{array}{c} \lim_{|z|\to \infty }|\theta_{z}\left(\gamma_{0}\right)-\theta |\leq \lim_{|z|\to \infty }|\theta_{z}\left(\gamma_{0}\right)-\theta \left(\gamma_{0}\right)|+\lim_{|z|\to \infty }\left|\theta \left(\gamma_{0}\right)-\theta \right|. \end{array}$$

We will show that the first term of RHS of (33) is equal to zero. Suppose that it is not zero, without loss of generality, by going to a subsequence if necessary, we may assume that $\theta_{z}\to \theta_{1}\neq \theta$ as |z|→∞. Since $\theta_{z}\left(\gamma_{0}\right)$ minimizes $HD^{2}\left(h_{J}\left(\cdot |\theta ,\gamma_{0}\right),h_{J}\left(\cdot |\theta ,\gamma_{0}\right)\right)$, it follows that

(34) $$\begin{array}{c} HD^{2}\left(h_{J,\alpha ,z}\left(\cdot |\theta ,\gamma_{0}\right),h_{J}\left(\cdot |\theta_{z},\gamma_{0}\right)\right)\leq HD^{2}\left(h_{J,\alpha ,z}\left(\cdot |\theta ,\gamma_{0}\right),h_{J}\left(\cdot |\theta^{\prime },\gamma_{0}\right)\right) \end{array}$$

for every $\theta^{\prime }\in \Theta$. We now show that as |z|→∞,

(35) $$\begin{array}{c} HD^{2}\left(h_{J,\alpha ,z}\left(\cdot |\theta ,\gamma_{0}\right),h_{J}\left(\cdot |\theta_{z},\gamma_{0}\right)\right)\to HD^{2}\left(\left(1-\alpha \right)h_{J}\left(\cdot |\theta ,\gamma_{0}\right),h_{J}\left(\cdot |\theta_{1},\gamma_{0}\right)\right). \end{array}$$

To this end, note that as |z|→∞, for every *y*,

$$\begin{array}{c} h_{J,\alpha ,z}\left(y|\theta ,\gamma_{0}\right)\to \left(1-\alpha \right)h_{J}\left(y|\theta ,\gamma_{0}\right),\;\mathrm{and}\;h_{J}\left(y|\theta_{z},\gamma_{0}\right)\to h_{J}\left(y|\theta_{1},\gamma_{0}\right) \end{array}$$

Therefore, as |z|→∞,

$$\begin{array}{ccc} \left|HD^{2}\left(h_{J,\alpha ,z}\left(\cdot |\theta ,\gamma_{0}\right),h_{J}\left(\cdot |\theta_{z},\gamma_{0}\right)\right)-HD^{2}\left(\left(1-\alpha \right)h_{J}\left(\cdot |\theta ,\gamma_{0}\right),h_{J}\left(\cdot |\theta_{1},\gamma_{0}\right)\right)\right| & \leq & 2(Q_{1}+Q_{2}), \end{array}$$

where

$$\begin{array}{c} Q_{1}=\int_{I\;R}\left|h_{J,\alpha ,z}^{\frac{1}{2}}\left(y|\theta ,\gamma_{0}\right)-{\left(\left(1-\alpha \right)h_{J}\left(y|\theta ,\gamma_{0}\right)\right)}^{\frac{1}{2}}\right|{\left(h_{J}\left(y|\theta_{z},\gamma_{0}\right))\right)}^{\frac{1}{2}}dy, \end{array}$$

$$\begin{array}{c} Q_{2}=\int_{I\;R}\left|h_{J}^{\frac{1}{2}}\left(y|\theta_{z},\gamma_{0}\right)-{\left(h_{J}\left(y|\theta_{1},\gamma_{0}\right)\right)}^{\frac{1}{2}}\right|{\left(\left(1-\alpha \right)h_{J}\left(y|\theta ,\gamma_{0}\right)\right)}^{\frac{1}{2}}dy. \end{array}$$

Now, by Cauchy-Schwarz inequality and Scheffe’s theorem, it follows that as |z|→∞, $Q_{1}\to 0$ and $Q_{2}\to 0$. Therefore, (35) holds. By Equations (34) and (35), we have

(36) $$\begin{array}{c} HD^{2}\left(\left(1-\alpha \right)h_{J}\left(\cdot |\theta ,\gamma_{0}\right),h_{J}\left(\cdot |\theta_{1},\gamma_{0}\right)\right)\leq HD^{2}\left(\left(1-\alpha \right)h_{J}\left(\cdot |\theta ,\gamma_{0}\right),h_{J}\left(\cdot |\theta^{\prime },\gamma_{0}\right)\right) \end{array}$$

for every $\theta^{\prime }\in \Theta$. Now consider

$$\begin{array}{c} HIF\left(\alpha ,h_{J}\left(\cdot |\theta ,\gamma_{0}\right),h_{J}\left(\cdot |\theta^{\prime },\gamma_{0}\right)\right)\equiv \int_{I\;R}{\left({\left[\left(1-\alpha \right)\delta (h_{J}\left(\cdot |\theta ,\gamma_{0}\right),h_{J}\left(y|\theta^{\prime },\gamma_{0}\right))+1\right]}^{\frac{1}{2}}-1\right)}^{2}h_{J}\left(y|\theta^{\prime },\gamma_{0}\right)dy, \end{array}$$

where $\delta \left(h_{J}\left(\cdot |\theta ,\gamma_{0}\right),h_{J}\left(y|\theta^{\prime },\gamma_{0}\right)\right)=\frac{h_{J}\left(y|\theta ,\gamma_{0}\right)}{h_{J}\left(y|\theta^{\prime },\gamma_{0}\right)}-1$. Since $G^{*}\left(\delta \right)={\left[{\left((1-\alpha )\delta +1\right)}^{\frac{1}{2}}-1\right]}^{2}$ is a non-negative and strictly convex function with δ=0 as the unique point of minimum. Hence $HIF(\alpha ,h_{J}\left(\cdot |\theta ,\gamma_{0}\right),h_{J}\left(\cdot |\theta^{\prime },\gamma_{0}\right))>0$ unless $\delta (h_{J}\left(\cdot |\theta ,\gamma_{0}\right),h_{J}\left(y|\theta^{\prime },\gamma_{0}\right))=0$ on a set of Lebesgue measure zero, which by the model identifiability assumption , is true if and only if $\theta^{\prime }=\theta$. Since $\theta_{1}\neq \theta$, it follows that

$$\begin{array}{c} HIF\left(\alpha ,h_{J}\left(\cdot |\theta ,\gamma_{0}\right),h_{J}\left(\cdot |\theta_{1},\gamma_{0}\right)\right)>HIF\left(\alpha ,h_{J}\left(\cdot |\theta ,\gamma_{0}\right),h_{J}\left(\cdot |\theta ,\gamma_{0}\right)\right). \end{array}$$

Since $HIF\left(\alpha ,h_{J}\left(\cdot |\theta ,\gamma_{0}\right),h_{J}\left(\cdot |\theta^{\prime },\gamma_{0}\right)\right)=HD^{2}\left(\left(1-\alpha \right)h_{J}\left(\cdot |\theta ,\gamma_{0}\right),h_{J}\left(\cdot |\theta^{\prime },\gamma_{0}\right)\right)-\alpha$. This implies that

$$\begin{array}{c} HD^{2}\left(\left(1-\alpha \right)h_{J}\left(\cdot |\theta ,\gamma_{0}\right),h_{J}\left(\cdot |\theta_{1},\gamma_{0}\right)\right)>HD^{2}\left(\left(1-\alpha \right)h_{J}\left(\cdot |\theta ,\gamma_{0}\right),h_{J}\left(\cdot |\theta^{\prime },\gamma_{0}\right)\right), \end{array}$$

which contradicts (36). The continuity of $\theta_{z}$ follows from Proposition 2 and the boundedness follows from the compactness of Θ. Now let $\gamma_{0}\to 0$, the second term of RHS of (33) converges to zero by Proposition 2.

We now turn to part (ii) of the Theorem. First fix $\gamma_{0}\geq 0.$ Since $\theta_{z}$ minimizes $H^{2}\left(h_{J,\alpha ,z}\left(\cdot |\theta ,\gamma_{0}\right),t\left(\gamma_{0}\right)\right)$ over Θ. By Taylor expansion of $HD^{2}\left(h_{J,\alpha ,z}\left(\cdot |\theta ,\gamma_{0}\right),h_{J}\left(\cdot |\theta_{z},\gamma_{0}\right)\right)$ around θ, we get

$$\begin{array}{ccc} 0=\nabla HD^{2}\left(h_{J,\alpha ,z}\left(\cdot |\theta ,\gamma_{0}\right),h_{J}\left(\cdot |\theta_{z},\gamma_{0}\right)\right) & = & HD^{2}\left(h_{J,\alpha ,z}\left(\cdot |\theta ,\gamma_{0}\right),h_{J}\left(\cdot |\theta ,\gamma_{0}\right)\right)\\ & \; & +\left(\theta_{z}-\theta \right)D\left(h_{J,\alpha ,z}\left(\cdot |\theta ,\gamma_{0}\right),h_{J}\left(\cdot |\theta_{z}^{*},\gamma_{0}\right)\right), \end{array}$$

where $\theta_{z}^{*}\left(\gamma_{0}\right)$ is a point between θ and $\theta_{z}$,

(37) $$\begin{array}{c} \nabla HD^{2}\left(h_{J,\alpha ,z}\left(\cdot |\theta ,\gamma_{0}\right),h_{J}\left(\cdot |\theta ,\gamma_{0}\right)\right)=\frac{1}{2}\int_{I\;R}u_{J,\alpha ,z}\left(y|\theta ,\gamma_{0}\right)s_{J,\alpha ,z}\left(y|\theta ,\gamma_{0}\right)\left(s_{J,\alpha ,z}\left(y|\theta ,\gamma_{0}\right)-s_{J}\left(y|\theta ,\gamma_{0}\right)\right)dy, \end{array}$$

and $D(h_{J,\alpha ,z}\left(\cdot |\theta ,\gamma_{0}\right),h_{J}\left(\cdot |\theta^{\prime },\gamma_{0}\right))$ can be expressed the sum of $D_{1}\left(h_{J,\alpha ,z}\left(\cdot |\theta ,\gamma_{0}\right),h_{J}\left(\cdot |\theta^{\prime },\gamma_{0}\right)\right)$ and $D_{2}\left(h_{J,\alpha ,z}\left(\cdot |\theta ,\gamma_{0}\right),h_{J}\left(\cdot |\theta^{\prime },\gamma_{0}\right)\right)$, where

(38) $$\begin{array}{c} D_{1}\left(h_{J,\alpha ,z}\left(\cdot |\theta ,\gamma_{0}\right),h_{J}\left(\cdot |\theta^{\prime },\gamma_{0}\right)\right)=\frac{1}{2}\int_{I\;R}{\dot{u}}_{J,\alpha ,z}\left(y|\theta ,\gamma_{0}\right)s_{J,\alpha ,z}\left(y|\theta ,\gamma_{0}\right)s_{J}\left(y|\theta^{\prime },\gamma_{0}\right)dy\;\mathrm{and} \end{array}$$

(39) $$\begin{array}{c} D_{2}\left(h_{J,\alpha ,z}\left(\cdot |\theta ,\gamma_{0}\right),h_{J}\left(\cdot |\theta^{\prime },\gamma_{0}\right)\right)=\frac{1}{4}\int_{I\;R}u_{J,\alpha ,z}\left(y|\theta ,\gamma_{0}\right)u_{J,\alpha ,z}^{\prime }\left(y|\theta ,\gamma_{0}\right)s_{J,\alpha ,z}\left(y|\theta ,\gamma_{0}\right)s_{J}\left(y|\theta^{\prime },\gamma_{0}\right)dy. \end{array}$$

Therefore,

$$\begin{array}{c} \alpha^{-1}\left(\theta_{z}-\theta \right)=-\alpha^{-1}D^{-1}\left(h_{J,\alpha ,z}\left(\cdot |\theta ,\gamma_{0}\right),h_{J}\left(\cdot |\theta_{z}^{*},\gamma_{0}\right)\right)\nabla HD^{2}\left(h_{J,\alpha ,z}\left(\cdot |\theta ,\gamma_{0}\right),h_{J}\left(\cdot |\theta ,\gamma_{0}\right)\right). \end{array}$$

We will show that

(40) $$\begin{array}{c} \lim_{\alpha \to 0}D\left(h_{J,\alpha ,z}\left(\cdot |\theta ,\gamma_{0}\right),h_{J}\left(\cdot |\theta_{z}^{*},\gamma_{0}\right)\right)=-\frac{1}{4}I\left(\theta \left(\gamma_{0}\right)\right),\;\mathrm{and} \end{array}$$

(41) $$\begin{array}{c} \lim_{\alpha \to 0}\alpha^{-1}\nabla HD^{2}\left(h_{J,\alpha ,z}\left(\cdot |\theta ,\gamma_{0}\right),h_{J}\left(\cdot |\theta ,\gamma_{0}\right)\right)=\frac{1}{4}\int_{I\;R}\left[\eta_{z}\left(y\right)u_{J}\left(y|\theta ,\gamma_{0}\right)\right]dy. \end{array}$$

We will first establish (40). Please note that as α→0, by definition $\theta_{z}\left(\alpha \right)\to \theta .$ Thus, $\lim_{\alpha \to 0}\theta_{z}^{*}\left(\alpha \right)=\theta .$ In addition, by assumptions (**O3**) and (**O4**), $D(h_{J,\alpha ,z}\left(\cdot |\theta ,\gamma_{0}\right),h_{J}\left(\cdot |\theta_{z},\gamma_{0}\right))$ is continuous in $\theta_{z}$. Therefore, to prove (40), it suffices to show that

(42) $$\begin{array}{c} \lim_{\alpha \to 0}D_{1}\left(h_{J,\alpha ,z}\left(\cdot |\theta ,\gamma_{0}\right),h_{J}\left(\cdot |\theta ,\gamma_{0}\right)\right)=-\frac{1}{2}\int_{I\;R}{\dot{u}}_{J,\alpha ,z}\left(y|\theta ,\gamma_{0}\right)h_{J}\left(y|\theta ,\gamma_{0}\right)dy=-\frac{1}{2}I\left(\theta \left(\gamma_{0}\right)\right),\;\mathrm{and} \end{array}$$

(43) $$\begin{array}{c} \lim_{\alpha \to 0}D_{2}\left(h_{J,\alpha ,z}\left(\cdot |\theta ,\gamma_{0}\right),h_{J}\left(\cdot |\theta ,\gamma_{0}\right)\right)=\frac{1}{4}\int_{I\;R}u_{J,\alpha ,z}\left(y|\theta ,\gamma_{0}\right)u_{J,\alpha ,z}^{\prime }\left(y|\theta ,\gamma_{0}\right)h_{J}\left(y|\theta ,\gamma_{0}\right)dy=\frac{1}{4}I\left(\theta \left(\gamma_{0}\right)\right). \end{array}$$

We begin with $D_{2}\left(h_{J,\alpha ,z}\left(\cdot |\theta ,\gamma_{0}\right),h_{J}\left(\cdot |\theta ,\gamma_{0}\right)\right)$. Notice that

$$\begin{array}{c} \lim_{\alpha \to 0}s_{J,\alpha ,z}\left(y|\theta ,\gamma_{0}\right)=s_{J}\left(y|\theta ,\gamma_{0}\right),\;\lim_{\alpha \to 0}u_{J,\alpha ,z}\left(y|\theta ,\gamma_{0}\right)=u_{J}\left(y|\theta ,\gamma_{0}\right),\;\mathrm{and} \end{array}$$

$$\begin{array}{c} \lim_{\alpha \to 0}{\dot{u}}_{J,\alpha ,z}\left(y|\theta ,\gamma_{0}\right)={\dot{u}}_{J}\left(y|\theta ,\gamma_{0}\right). \end{array}$$

Thus,

$$\begin{array}{c} \lim_{\alpha \to 0}{\dot{u}}_{J,\alpha ,z}\left(y|\theta ,\gamma_{0}\right)s_{J,\alpha ,z}\left(y|\theta ,\gamma_{0}\right)s_{J}\left(y|\theta ,\gamma_{0}\right)={\dot{u}}_{J}\left(y|\theta ,\gamma_{0}\right)h_{J}\left(y|\theta ,\gamma_{0}\right). \end{array}$$

In addition, in order to pass the limit inside the integral, note that, for every component of matrix $u_{J,\alpha ,z}\left(\cdot |\theta ,\gamma_{0}\right)u_{J,\alpha ,z}^{\prime }\left(\cdot |\theta ,\gamma_{0}\right)$, we have

$$\begin{array}{ccc} \left|u_{J,\alpha ,z}\left(y|\theta ,\gamma_{0}\right)u_{J,\alpha ,z}^{\prime }\left(y|\theta ,\gamma_{0}\right)\right| & = & \left|\left(\frac{\left(1-\alpha \right)\nabla h_{J,\alpha ,z}\left(y|\theta ,\gamma_{0}\right)}{\left(1-\alpha \right)h_{J,\alpha ,z}\left(y|\theta ,\gamma_{0}\right)+\alpha \eta_{z}\left(y\right)}\right){\left(\frac{\left(1-\alpha \right)\nabla h_{J,\alpha ,z}\left(y|\theta ,\gamma_{0}\right)}{\left(1-\alpha \right)h_{J,\alpha ,z}\left(y|\theta ,\gamma_{0}\right)+\alpha \eta_{z}\left(y\right)}\right)}^{\prime }\right|\\ & = & \left|\left(\frac{\nabla h_{J,\alpha ,z}\left(y|\theta ,\gamma_{0}\right)}{h_{J,\alpha ,z}\left(y|\theta ,\gamma_{0}\right)+\frac{\alpha }{1-\alpha }\eta_{z}\left(y\right)}\right){\left(\frac{\nabla h_{J,\alpha ,z}\left(y|\theta ,\gamma_{0}\right)}{h_{J,\alpha ,z}\left(y|\theta ,\gamma_{0}\right)+\frac{\alpha }{1-\alpha }\eta_{z}\left(y\right)}\right)}^{\prime }\right|\\ & \leq & \left|\left(\frac{\nabla h_{J,\alpha ,z}\left(y|\theta ,\gamma_{0}\right)}{h_{J,\alpha ,z}\left(y|\theta ,\gamma_{0}\right)}\right){\left(\frac{\nabla h_{J,\alpha ,z}\left(y|\theta ,\gamma_{0}\right)}{h_{J,\alpha ,z}\left(y|\theta ,\gamma_{0}\right)}\right)}^{\prime }\right|=\left|u_{J}\left(y|\theta ,\gamma_{0}\right)u_{J}^{\prime }\left(y|\theta ,\gamma_{0}\right)\right|, \end{array}$$

where |·| represents the absolute function for each component of the matrix, and

$$\begin{array}{c} \left|s_{J,\alpha ,z}\left(y|\theta ,\gamma_{0}\right)\right|\leq {\left[h_{J}\left(y|\theta ,\gamma_{0}\right)+\eta_{z}\left(y\right)\right]}^{\frac{1}{2}}. \end{array}$$

Now choosing the dominating function

$$\begin{array}{c} m_{J}^{\left(1\right)}\left(y|\theta ,\gamma_{0}\right)=\left|u_{J}\left(y|\theta ,\gamma_{0}\right)u_{J}^{\prime }\left(y|\theta ,\gamma_{0}\right)\right|{\left[h_{J}\left(y|\theta ,\gamma_{0}\right)+\eta_{z}\left(y\right)\right]}^{\frac{1}{2}}s_{J}\left(y|\theta ,\gamma_{0}\right) \end{array}$$

and applying Cauchy-Schwarz inequality, we obtain that there exists a constant *C* such that

$$\begin{array}{ccc} \int_{I\;R}\left|m_{J}^{\left(1\right)}\left(y|\theta ,\gamma_{0}\right)\right|dy & \leq & C{\left\{\int_{I\;R}{\left(u_{J}\left(y|\theta ,\gamma_{0}\right)u_{J}^{\prime }\left(y|\theta ,\gamma_{0}\right)s_{J}\left(y|\theta ,\gamma_{0}\right)\right)}^{2}dy\right\}}^{\frac{1}{2}}, \end{array}$$

which is finite by assumption (**M2**). Hence, by the dominated convergence theorem, (43) holds. Turning to (42), notice that for each component of the matrix ${\dot{u}}_{J,\alpha ,z}\left(y|\theta ,\gamma_{0}\right)$,

$$\begin{array}{ccc} \left|{\dot{u}}_{J,\alpha ,z}\left(y|\theta ,\gamma_{0}\right)\right| & = & \left|\frac{{\ddot{h}}_{J}\left(y|\theta ,\gamma_{0}\right)\left[h_{J}\left(y|\theta ,\gamma_{0}\right)+\frac{\alpha }{1-\alpha }\eta_{z}\left(y\right)\right]-\left(\nabla h_{J}\left(y|\theta ,\gamma_{0}\right)\right){\left(\nabla h_{J}\left(y|\theta ,\gamma_{0}\right)\right)}^{\prime }}{{\left(h_{J}\left(y|\theta ,\gamma_{0}\right)+\frac{\alpha }{1-\alpha }\eta_{z}\left(y\right)\right)}^{2}}\right|\\ & \leq & \left|\frac{{\ddot{h}}_{J}\left(y|\theta ,\gamma_{0}\right)}{h_{J}\left(y|\theta ,\gamma_{0}\right)}\right|+\left|\frac{\left(\nabla h_{J}\left(y|\theta ,\gamma_{0}\right)\right){\left(\nabla h_{J}\left(y|\theta ,\gamma_{0}\right)\right)}^{\prime }}{h_{J}^{2}\left(y|\theta ,\gamma_{0}\right)}\right|, \end{array}$$

where |·| denotes the absolute function for each component. Now choosing the dominating function

$$\begin{array}{c} m_{J}^{\left(2\right)}\left(y|\theta ,\gamma_{0}\right)=\left(\left|\frac{{\ddot{h}}_{J}\left(y|\theta ,\gamma_{0}\right)}{h_{J}\left(y|\theta ,\gamma_{0}\right)}\right|+\left|\frac{\left(\nabla h_{J}\left(y|\theta ,\gamma_{0}\right)\right){\left(\nabla h_{J}\left(y|\theta ,\gamma_{0}\right)\right)}^{\prime }}{h_{J}^{2}\left(y|\theta ,\gamma_{0}\right)}\right|\right){\left[h_{J}\left(y|\theta ,\gamma_{0}\right)+\eta_{z}\left(y\right)\right]}^{\frac{1}{2}}s_{J}\left(y|\theta ,\gamma_{0}\right), \end{array}$$

and applying the Cauchy-Schwarz inequality it follows, using (**M3**), that

$$\begin{array}{c} \int_{I\;R}\left|m_{J}^{\left(2\right)}\left(y|\theta ,\gamma_{0}\right)\right|dy<\infty . \end{array}$$

Finally, by the dominated convergence theorem, it follows that

$$\begin{array}{c} \lim_{\alpha \to 0}D_{1}\left(h_{J,\alpha ,z}\left(\cdot |\theta ,\gamma_{0}\right),h_{J}\left(\cdot |\theta_{z}^{*},\gamma_{0}\right)\right)=-\frac{1}{2}I\left(\theta \left(\gamma_{0}\right)\right). \end{array}$$

Therefore (40) follows. It remains to show that (41) holds. To this end, note that

$$\begin{array}{ccc} \nabla HD^{2}\left(h_{J,\alpha ,z}\left(\cdot |\theta ,\gamma_{0}\right),h_{J}\left(\cdot |\theta ,\gamma_{0}\right)\right) & = & -\frac{1}{2}\int_{I\;R}s_{J,\alpha ,z}\left(y|\theta ,\gamma_{0}\right)u_{J,\alpha ,z}\left(y|\theta ,\gamma_{0}\right)s_{J}\left(y|\theta ,\gamma_{0}\right)dy. \end{array}$$

Now taking partial derivative of $HD^{2}\left(h_{J,\alpha ,z}\left(\cdot |\theta ,\gamma_{0}\right),h_{J}\left(\cdot |\theta ,\gamma_{0}\right)\right)$ with respect to α, it can be expressed as the sum of $U_{1}$, $U_{2}$ and $U_{3}$, where

$$\begin{array}{c} U_{1}=-\frac{1}{4}\int_{I\;R}\frac{-h_{J}\left(y|\theta ,\gamma_{0}\right)+\eta_{z}\left(y\right)}{s_{J,\alpha ,z}\left(y|\theta ,\gamma_{0}\right)}u_{J,\alpha ,z}\left(y|\theta ,\gamma_{0}\right)s_{J}\left(y|\theta ,\gamma_{0}\right)dy, \end{array}$$

$$\begin{array}{c} U_{2}=-\frac{1}{2}\int_{I\;R}s_{J,\alpha ,z}\left(y|\theta ,\gamma_{0}\right)\frac{-\nabla h_{J}\left(y|\theta ,\gamma_{0}\right)h_{J,\alpha ,z}\left(y|\theta ,\gamma_{0}\right)}{h_{J,\alpha ,z}^{2}\left(y|\theta ,\gamma_{0}\right)}s_{J}\left(y|\theta ,\gamma_{0}\right)dy,\;\mathrm{and} \end{array}$$

$$\begin{array}{c} U_{3}=-\frac{1}{2}\int_{I\;R}s_{J,\alpha ,z}\left(y|\theta ,\gamma_{0}\right)\frac{-\left(1-\alpha \right)\nabla h_{J}\left(y|\theta ,\gamma_{0}\right)\left(-h_{J}\left(y|\theta ,\gamma_{0}\right)+\eta_{z}\left(y\right)\right)}{h_{J,\alpha ,z}^{2}\left(y|\theta ,\gamma_{0}\right)}s_{J}\left(y|\theta ,\gamma_{0}\right)dy. \end{array}$$

By dominated convergence theorem (using similar idea as above to find dominating functions), we have

$$\begin{array}{c} \lim_{\alpha \to 0}\frac{\partial \nabla HD^{2}\left(h_{J,\alpha ,z}\left(\cdot |\theta ,\gamma_{0}\right),h_{J}\left(\cdot |\theta ,\gamma_{0}\right)\right)}{\partial \alpha }=\frac{1}{4}\int_{I\;R}u_{J}\left(y|\theta ,\gamma_{0}\right)\eta_{z}\left(y\right)dy. \end{array}$$

Hence, by L’Hospital rule, (41) holds. It remains to show that

(44) $$\begin{array}{c} \lim_{\gamma_{0}\to 0}\lim_{\alpha \to 0}D\left(h_{J,\alpha ,z}\left(\cdot |\theta ,\gamma_{0}\right),h_{J}\left(\cdot |\theta ,\gamma_{0}\right)\right)=-\frac{1}{4}I\left(\theta \right),\;\mathrm{and} \end{array}$$

(45) $$\begin{array}{c} \lim_{\gamma_{0}\to 0}\lim_{\alpha \to 0}\alpha^{-1}\nabla HD^{2}\left(h_{J,\alpha ,z}\left(\cdot |\theta ,\gamma_{0}\right),h_{J}\left(\cdot |\theta ,\gamma_{0}\right)\right)=\frac{1}{4}\int_{I\;R}\left[\eta_{z}\left(y\right)v_{J}\left(y|\theta \right)\right]dy. \end{array}$$

We start with (44). Since for fixed $\gamma_{0}\geq 0$, by the above argument, it follows that

$$\begin{array}{c} \lim_{\alpha \to 0}D\left(h_{J,\alpha ,z}\left(\cdot |\theta ,\gamma_{0}\right),h_{J}\left(\cdot |\theta ,\gamma_{0}\right)\right)=-\frac{1}{4}I\left(\theta \left(\gamma_{0}\right)\right)=-\frac{1}{4}\int_{I\;R}u_{J}\left(y|\theta ,\gamma_{0}\right)u_{J}^{\prime }\left(y|\theta ,\gamma_{0}\right)h_{J}\left(y|\theta ,\gamma_{0}\right)dy, \end{array}$$

it is enough to show

$$\begin{array}{c} \lim_{\gamma_{0}\to 0}\int_{I\;R}u_{J}\left(y|\theta ,\gamma_{0}\right)u_{J}^{\prime }\left(y|\theta ,\gamma_{0}\right)h_{J}\left(y|\theta ,\gamma_{0}\right)dy=\int_{I\;R}v_{J}\left(y|\theta \right)v_{J}^{\prime }\left(y|\theta \right)h^{*J}\left(y|\theta \right)dy, \end{array}$$

which is proved in Lemma 2. Hence (44) holds. Next we prove (45). By the argument used to establish (40), it is enough to show that

(46) $$\begin{array}{c} \lim_{\gamma_{0}\to 0}\int_{I\;R}\left[\eta_{z}\left(y\right)u_{J}\left(y|\theta ,\gamma_{0}\right)\right]dy=\int_{I\;R}\left[\eta_{z}\left(y\right)v_{J}\left(y|\theta \right)\right]dy. \end{array}$$

However,

$$\begin{array}{c} \int_{I\;R}\eta_{z}\left(y\right)\left[u_{J}\left(y|\theta ,\gamma_{0}\right)-v_{J}\left(y|\theta \right)\right]dy\leq \underset{y}{\sup}\left|u_{J}\left(y|\theta ,\gamma_{0}\right)-v_{J}\left(y|\theta \right)\right|, \end{array}$$

and the RHS of the above inequality converges to zero as $\gamma_{0}\to 0$ from assumption (**V1**). Hence (46) holds. This completes the proof. □

Our next result is concerned with the behavior of the α−influence function when $\gamma_{0}\to 0$ first and then |z|→∞ or α→0. The following three additional assumptions will be used in the proof of part (ii) of Theorem 4.

**Model assumptions for robustness analysis**

(**O5**) For α∈[0,1] and all θ∈Θ, ${\dot{u}}_{\alpha ,z}^{*J}\left(y|\theta \right)s_{\alpha ,z}^{*J}\left(y|\theta \right)$ is bounded in $L_{2}$.

(**O6**) For α∈[0,1] and all θ∈Θ, $u_{\alpha ,z}^{*J}\left(y|\theta \right)u_{\alpha ,z}^{*J^{\prime }}\left(y|\theta \right)s_{\alpha ,z}^{*J}\left(y|\theta \right)$ is bounded in $L_{2}$.

(**O7**) For α∈[0,1] and all θ∈Θ,

$$\begin{array}{c} \lim_{\gamma_{0}\to 0}\int_{I\;R}{\left(s_{J,\alpha ,z}\left(y|\theta ,\gamma_{0}\right)u_{J,\alpha ,z}\left(y|\theta ,\gamma_{0}\right)-s_{\alpha ,z}^{*J}\left(y|\theta \right)u_{\alpha ,z}^{*J}\left(y|\theta \right)\right)}^{2}dy=0. \end{array}$$

**Theorem** **4.** *(i) Let α∈(0,1), and assume that for all θ∈Θ, assume that the assumptions of Proposition 1 hold, also assume that $T(h_{J,\alpha ,z}\left(\cdot |\theta ,\gamma_{0}\right))$ is unique for all z. Then, $T(h_{J,\alpha ,z}\left(\cdot |\theta ,\gamma_{0}\right))$ is a bounded, continuous function of z such that*

$$\begin{array}{c} \lim_{|z|\to \infty }\lim_{\gamma_{0}\to 0}T\left(h_{J,\alpha ,z}\left(\cdot |\theta ,\gamma_{0}\right)\right)=\theta ; \end{array}$$

*(ii) Assume further that the conditions (**O3**)–(**O7**) hold. Then,*

$$\begin{array}{c} \lim_{\alpha \to 0}\lim_{\gamma_{0}\to 0}\alpha^{-1}\left[T(h_{J,\alpha ,z}\left(\cdot |\theta ,\gamma_{0}\right))-\theta \right]={\left[I(\theta )\right]}^{-1}\int_{I\;R}\left[\eta_{z}\left(y\right)v_{J}\left(y|\theta \right)\right]dy. \end{array}$$

**Proof.**  Let $\theta_{z}\left(\gamma_{0}\right)$ denote $T(h_{J,\alpha ,z}\left(\cdot |\theta ,\gamma_{0}\right))$ and let $\theta_{z}$ denote $T(h_{\alpha ,z}^{*J}\left(\cdot |\theta \right))$. First fix z∈IR; then by the triangular inequality,

(47) $$\begin{array}{c} \lim_{\gamma_{0}\to 0}|\theta_{z}\left(\gamma_{0}\right)-\theta |\leq \lim_{\gamma_{0}\to 0}|\theta_{z}\left(\gamma_{0}\right)-\theta_{z}|+\lim_{\gamma_{0}\to 0}\left|\theta_{z}-\theta \right|. \end{array}$$

The first term of RHS of (47) is equal to zero due to proposition 2. Now let |z|→∞, then the second term on the RHS of (47) converges to zero using similar argument as Theorem 3 with density converging to $h_{\alpha ,z}^{*J}\left(\cdot |\theta \right)$. This completes the proof of I). Turning to (ii), we will prove that

(48) $$\begin{array}{c} \lim_{\alpha \to 0}\lim_{\gamma_{0}\to 0}D\left(h_{J,\alpha ,z}\left(\cdot |\theta ,\gamma_{0}\right),h_{J}\left(\cdot |\theta ,\gamma_{0}\right)\right)=-\frac{1}{4}I\left(\theta \right), \end{array}$$

(49) $$\begin{array}{c} \lim_{\alpha \to 0}\lim_{\gamma_{0}\to 0}\alpha^{-1}\nabla HD^{2}\left(h_{J,\alpha ,z}\left(\cdot |\theta ,\gamma_{0}\right),h_{J}\left(\cdot |\theta ,\gamma_{0}\right)\right)=\frac{1}{4}\int_{I\;R}\left[\eta_{z}\left(y\right)v_{J}\left(y|\theta \right)\right]dy. \end{array}$$

Recall from the proof of part (ii) of Theorem 3 that

$$\begin{array}{ccc} D(h_{J,\alpha ,z}\left(\cdot |\theta ,\gamma_{0}\right),h_{J}\left(\cdot |\theta ,\gamma_{0}\right)) & = & \frac{1}{2}\int_{I\;R}{\dot{u}}_{J,\alpha ,z}\left(y|\theta ,\gamma_{0}\right)s_{J,\alpha ,z}\left(y|\theta ,\gamma_{0}\right)s_{J}\left(y|\theta ,\gamma_{0}\right)\\ & \; & +\frac{1}{4}\int_{I\;R}u_{J,\alpha ,z}\left(y|\theta ,\gamma_{0}\right)u_{J,\alpha ,z}^{\prime }\left(y|\theta ,\gamma_{0}\right)s_{J,\alpha ,z}\left(y|\theta ,\gamma_{0}\right)s_{J}\left(y|\theta ,\gamma_{0}\right)\\ & \equiv & D_{1}\left(h_{J,\alpha ,z}\left(\cdot |\theta ,\gamma_{0}\right),h_{J}\left(\cdot |\theta ,\gamma_{0}\right)\right)+D_{2}\left(h_{J,\alpha ,z}\left(\cdot |\theta ,\gamma_{0}\right),h_{J}\left(\cdot |\theta ,\gamma_{0}\right)\right). \end{array}$$

We will now show that for fixed α∈(0,1)

(50) $$\begin{array}{c} \lim_{\gamma_{0}\to 0}D_{1}\left(h_{J,\alpha ,z}\left(\theta ,\gamma_{0}\right),h_{J}\left(\cdot |\theta ,\gamma_{0}\right)\right)=\frac{1}{2}\int_{I\;R}{\dot{u}}_{\alpha ,z}^{*J}\left(y|\theta \right)s_{\alpha ,z}^{*J}\left(y|\theta \right)t_{J}\left(y|\theta \right)dy,\;\mathrm{and} \end{array}$$

(51) $$\begin{array}{c} \lim_{\gamma_{0}\to 0}D_{2}\left(h_{J,\alpha ,z}\left(\theta ,\gamma_{0}\right),h_{J}\left(\cdot |\theta ,\gamma_{0}\right)\right)=\frac{1}{4}\int_{I\;R}u_{\alpha ,z}^{*J}\left(y|\theta \right)u_{\alpha ,z}^{*J^{\prime }}\left(y|\theta \right)s_{\alpha ,z}^{*J}\left(y|\theta \right)t_{J}\left(y|\theta \right)dy. \end{array}$$

We begin with (50). A standard calculation shows that $D_{1}\left(h_{J,\alpha ,z}\left(\theta ,\gamma_{0}\right),u_{\alpha ,z}^{*J}\left(y|\theta \right)\right)$ can be expressed as the sum of $D_{11}$, $D_{12}$ and $D_{13}$, where

$$\begin{array}{c} D_{11}=\frac{1}{2}\int_{I\;R}\left({\dot{u}}_{J,\alpha ,z}\left(y|\theta ,\gamma_{0}\right)s_{J,\alpha ,z}\left(y|\theta ,\gamma_{0}\right)-{\dot{u}}_{\alpha ,z}^{*J}\left(y|\theta \right)s_{\alpha ,z}^{*J}\left(y|\theta \right)\right)s_{J}\left(y|\theta ,\gamma_{0}\right)dy, \end{array}$$

$$\begin{array}{c} D_{12}=\frac{1}{2}\int_{I\;R}{\dot{u}}_{\alpha ,z}^{*J}\left(y|\theta \right)s_{\alpha ,z}^{*J}\left(y|\theta \right)\left(s_{J}\left(y|\theta ,\gamma_{0}\right)-t_{J}\left(y|\theta \right)\right)dy,\;\mathrm{and} \end{array}$$

$$\begin{array}{c} D_{13}=\frac{1}{2}\int_{I\;R}{\dot{u}}_{\alpha ,z}^{*J}\left(y|\theta \right)s_{\alpha ,z}^{*J}\left(y|\theta \right)t_{J}\left(y|\theta \right)dy. \end{array}$$

It can be seen that $D_{11}$ converges to zero as $\gamma_{0}\to 0$ by Cauchy-Schwarz inequality and assumption (**O3**); also, $D_{12}$ converges to zero as $\gamma_{0}\to 0$ by Cauchy-Schwarz inequality, assumption (**O5**) and Scheffe’s theorem. Hence (50) follows. Similarly (51) follows as $\gamma_{0}\to 0$ by Cauchy-Schwarz inequality, assumption (**O4**), assumption (**O6**) and Scheffe’s theorem.

Now let α→0. Using the same idea as in Theorem 3 to find dominating functions, one can apply the dominated convergence Theorem to establish that

$$\begin{array}{c} \lim_{\alpha \to 0}\frac{1}{2}\int_{I\;R}{\dot{u}}_{\alpha ,z}^{*J}\left(y|\theta \right)s_{\alpha ,z}^{*J}\left(y|\theta \right)t_{J}\left(y|\theta \right)dy=-\frac{1}{2}I\left(\theta \right),\;\mathrm{and} \end{array}$$

$$\begin{array}{c} \lim_{\alpha \to 0}\frac{1}{4}\int_{I\;R}u_{\alpha ,z}^{*J}\left(y|\theta \right)u_{\alpha ,z}^{*J^{\prime }}\left(y|\theta \right)s_{\alpha ,z}^{*J}\left(y|\theta \right)t_{J}\left(y|\theta \right)dy=\frac{1}{4}I\left(\theta \right). \end{array}$$

Hence (48) follows. Finally, it remains to establish (49). First fix α∈(0,1); we will show that

(52) $$\begin{array}{c} \lim_{\gamma_{0}\to 0}\nabla HD^{2}\left(h_{J,\alpha ,z}\left(\cdot |\theta ,\gamma_{0}\right),h_{J}\left(\cdot |\theta ,\gamma_{0}\right)\right)=-\frac{1}{2}\int_{I\;R}s_{\alpha ,z}^{*J}\left(y|\theta \right)u_{\alpha ,z}^{*J}\left(y|\theta \right)t_{J}\left(y|\theta \right)dy. \end{array}$$

Please $\nabla HD^{2}\left(h_{J,\alpha ,z}\left(\cdot |\theta ,\gamma_{0}\right),h_{J}\left(\cdot |\theta ,\gamma_{0}\right)\right)$ can be expressed as the sum of $T_{1}$, $T_{2}$ and $T_{3}$, where

$$\begin{array}{c} T_{1}=-\frac{1}{2}\int_{I\;R}\left(s_{J,\alpha ,z}\left(y|\theta ,\gamma_{0}\right)u_{J,\alpha ,z}\left(y|\theta ,\gamma_{0}\right)-s_{\alpha ,z}^{*J}\left(y|\theta \right)u_{\alpha ,z}^{*J}\left(y|\theta \right)\right)s_{J}\left(y|\theta ,\gamma_{0}\right)dy, \end{array}$$

$$\begin{array}{c} T_{2}=-\frac{1}{2}\int_{I\;R}s_{\alpha ,z}^{*J}\left(y|\theta \right)u_{\alpha ,z}^{*J}\left(y|\theta \right)\left(s_{J}\left(y|\theta ,\gamma_{0}\right)-t_{J}\left(y|\theta \right)\right)dy,\;\mathrm{and} \end{array}$$

$$\begin{array}{c} T_{3}=-\frac{1}{2}\int_{I\;R}s_{\alpha ,z}^{*J}\left(y|\theta \right)u_{\alpha ,z}^{*J}\left(y|\theta \right)t_{J}\left(y|\theta \right)dy. \end{array}$$

It can be seen thatr $T_{1}$ converges to zero as $\gamma_{0}\to 0$ by Cauchy-Schwarz inequality and assumption (**O7**); $T_{2}$ converges to zero as $\gamma_{0}\to 0$ by Cauchy-Schwarz inequality, boundedness of $u_{\alpha ,z}^{*J}\left(\cdot \right)s_{\alpha ,z}^{*J}\left(\cdot \right)$ in $L_{2}$, and Scheffe’s theorem. Therefore, (52) holds. Finally, letting α→0 and using the same idea as in Theorem 3 to find the dominating function, it follows by the dominated convergence theorem and L’Hospital rule that (49) holds. This completes the proof of the Theorem. □

**Remark** **4.** *Theorems 3 and 4 do not imply that the double limit exists. This is beyond the scope of this paper.*

In the next section, we describe the implementation details and provide several simulation results in support of our methodology.

## 4. Implementation and Numerical Results

In this section, we apply the proposed MHD based methods to estimate the unknown parameters $\theta =(\mu ,\sigma^{2})$ using the compressed data. We set *J* = 10,000 and B=100. All simulations are based on 5000 replications. We consider the Gaussian kernel and Epanechnikov kernel for the nonparametric density estimation. The Gaussian kernel is given by

$$\begin{array}{c} K\left(x\right)=\frac{1}{\sqrt{2\pi }}\exp\left(-\frac{x^{2}}{2}\right), \end{array}$$

and the Epanechnikov kernel is given by

$$\begin{array}{c} K\left(x\right)=\frac{3}{4}\left(1-x^{2}\right)1_{\left(|x|\leq 1\right)}. \end{array}$$

We generate X and uncontaminated compressed data $\tilde{Y}$ in the following way:

- Step 1. Generate $X_{l}$, where $X_{jl}\overset{i.i.d.}{∼}N\left(\mu ,\sigma^{2}\right)$.
- Step 2. Generate $R_{l}$, where $r_{ijl}\overset{i.i.d.}{∼}N\left(1,\gamma_{0}^{2}\right)$.
- Step 3. Generate the uncontaminated ${\tilde{Y}}_{l}$ by calculating ${\tilde{Y}}_{l}=R_{l}X_{l}$.

### 4.1. Objective Function

In practice, we store the compressed data $\left({\tilde{Y}}_{l},r_{\cdot l},\omega_{l}\right)$ for all 1≤l≤B. Hence if $X_{jl}$ follows Normal distribution with mean μ and variance $\sigma^{2}$, the form of the marginal density of the compressed data, *viz.,* $Y_{il}$ is complicated and does not have a closed form expression. However, for large *J*, using the local limit theorem its density can be approximated by Gaussian density with mean $\sqrt{J}\mu$ and variance $\sigma^{2}+\gamma_{0}^{2}\left(\mu^{2}+\sigma^{2}\right)$. Hence, we work with $U_{il}$, where $U_{il}=\frac{{\tilde{Y}}_{il}-\mu r_{i\cdot l}}{\omega_{il}}$. Please note that with this transformation, $E[U_{il}]=0$ and $\mathrm{Var}\left[U_{il}\right]=\sigma^{2}$. Hence, the kernel density estimate of the unknown true density is given by

$$\begin{array}{c} g_{B}^{\left(i\right)}\left(y|\mu \right)=\frac{1}{Bc_{B}}\sum_{l=1}^{B}K\left(\frac{y-U_{il}}{c_{B}}\right). \end{array}$$

The difference between the kernel density estimate and the one proposed here is that we include the unknown parameter μ in the kernel. Additionally, this allows one to incorporate $\left(r_{\cdot r},\omega_{l}\right)$ into the kernel. Consequently, only the scale parameter σ is part of the parametric model. Using the local limit theorem, we approximate the true parametric model by ϕ(·|σ), where ϕ(·|σ) is the density of $N(0,\sigma^{2})$. Hence, the objective function is

$$\begin{array}{c} \Psi \left(i,\theta \right)\equiv A(g_{B}^{\left(i\right)}\left(\cdot |\mu ),\phi (\cdot |\sigma )\right)=\int_{I\;R}g_{B}^{\left(i\right)\frac{1}{2}}\left(y|\mu \right)\phi^{\frac{1}{2}}\left(y|\sigma \right)dy; \end{array}$$

and, the estimator is given by

$$\begin{array}{c} {\hat{\theta }}_{B}\left(\gamma_{0}\right)=\frac{1}{S}\sum_{i=1}^{S}{\hat{\theta }}_{i,B}\left(\gamma_{0}\right),\;\mathrm{where}\;{\hat{\theta }}_{i,B}\left(\gamma_{0}\right)=\underset{\theta \in \Theta }{\mathrm{argmax}}\;\Psi \left(i,\theta \right). \end{array}$$

It is clear that ${\hat{\theta }}_{B}\left(\gamma_{0}\right)$ is a consistent estimator of $\theta^{*}.$ In the next subsection, we use Quasi-Newton method with Broyden-Fletcher-Goldfarb-Shanno (BFGS) update to estimate θ. Quasi-Newton method is appealing since (i) it replaces the complicated calculation of the Hessian matrix with an approximation which is easier to compute ($\Delta_{k}\left(\theta \right)$ given in the next subsection) and (ii) gives more flexible step size *t* (compared to the Newton-Raphson method), ensuring that it does not “jump” too far at every step and hence guaranteeing convergence of the estimating equation. The BFGS update ($H_{k}$) is a popular method for approximating the Hessian matrix via gradient evaluations. The step size *t* is determined using Backtracking line search algorithm described in Algorithm 2. The algorithms are given in detail in the next subsection. Our analysis also includes the case where S≡1 and $r_{ijl}\equiv 1$. In this case, as explained previously, one obtains significant reduction in storage and computational complexity. Finally, we emphasize here that the density estimate contains μ and is not parameter free as is typical in classical MHDE analysis. In the next subsection, we describe an algorithm to implement our method.

### 4.2. Algorithm

As explained previously, we use the Quasi-Newton Algorithm with BFGS update to obtain ${\hat{\theta }}_{\mathrm{MHDE}}$. To describe this method, consider the objective function (suppressing *i*) Ψ(θ), which is twice continuously differentiable. Let the initial value of θ be $\theta^{\left(0\right)}=\left(\mu^{\left(0\right)},\sigma^{\left(0\right)}\right)$ and $H_{0}=I$, where *I* is the identity matrix.

**Algorithm 1:** The Quasi-Newton Algorithm.Set k = 1. **repeat**Calculate $\Delta_{k}\left(\theta \right)=-H_{k-1}^{-1}\nabla \Psi \left(\theta^{\left(k-1\right)}\right)$, where $\nabla \Psi (y;\theta^{k-1})$ is the first derivative of Ψ(θ) with respect to θ at (k−1)th step.Determine the step length parameter *t* via backtracking line search.Compute $\theta^{\left(k\right)}=\theta^{\left(k-1\right)}+t\Delta_{k}\left(\theta \right)$.Compute $H_{k}$, where the BFGS update is
$$\begin{array}{c} H_{k}=H_{k-1}+\frac{q_{k-1}q_{k-1}^{T}}{q_{k-1}^{T}d_{k-1}}-\frac{H_{k-1}d_{k-1}d_{k-1}^{T}H_{k-1}^{T}}{d_{k-1}^{T}H_{k-1}d_{k-1}}, \end{array}$$
where
$$\begin{array}{c} d_{k-1}=\theta^{\left(k\right)}-\theta^{\left(k-1\right)},\\ q_{k-1}=\nabla \Psi \left(\theta^{\left(k\right)}\right)-\nabla \Psi \left(\theta^{\left(k-1\right)}\right). \end{array}$$Compute $e_{k}=\left|\Psi \left(\theta^{\left(k\right)}\right)-\Psi \left(\theta^{\left(k-1\right)}\right)\right|$.Set k=k+1.**until**$(e_{k})<\mathrm{threshold}$.

**Remark** **5.** *In step 1, one can directly use the Inverse update for $H_{k}^{-1}$ as follows:*

$$\begin{array}{c} H_{k}^{-1}=\left(I-\frac{d_{k-1}q_{k-1}^{T}}{q_{k-1}^{T}d_{k-1}}\right)H_{k-1}^{-1}\left(I-\frac{q_{k-1}d_{k-1}^{T}}{q_{k-1}^{T}d_{k-1}}\right)+\frac{d_{k-1}d_{k-1}^{T}}{q_{k-1}^{T}d_{k-1}}. \end{array}$$

**Remark** **6.** *In step 2, the step size t should satisfy the Wolfe conditions:*

$$\begin{array}{c} \begin{array}{c} \Psi \left(y;\theta^{\left(k\right)}+t\Delta_{k}\right)\leq \Psi \left(\theta^{\left(k\right)}\right)+u_{1}t\nabla \Psi^{T}\left(\theta^{\left(k\right)}\right)\Delta_{k},\\ \nabla \Psi \left(\theta^{\left(k\right)}+t\Delta_{k}\right)\geq u_{2}\nabla \Psi^{T}\left(\theta^{\left(k\right)}\right)\Delta_{k}, \end{array} \end{array}$$

*where $u_{1}$ and $u_{2}$ are constants with $0<u_{1}<u_{2}<1.$ The first condition requires that t sufficiently decrease the objective function. The second condition ensures that the step size is not too small. The Backtracking line search algorithm proceeds as follows (see [26]):*

**Algorithm 2:** The Backtracking Line Search Algorithm.Given a descent direction Δ(θ) for Ψ at θ, ζ∈(0,0.5),κ∈(0,1). t:=1.     **while**
$\Psi \left(\theta +t\Delta \theta \right)>\Psi \left(\theta \right)+\zeta t\nabla \Psi {\left(\theta \right)}^{T}\Delta \theta$,    **do**        t:=κt.    **end while**

### 4.3. Initial Values

The initial value for θ are taken to be

$$\begin{array}{c} \begin{array}{c} \mu^{\left(0\right)}=\mathrm{median}\left({\tilde{Y}}_{il}\right)/J,\\ \sigma^{\left(0\right)}=1.48\times \mathrm{median}\left(|{\tilde{Y}}_{il}-\mathrm{median}\left({\tilde{Y}}_{il}\right)|\right)/B. \end{array} \end{array}$$

Another choice of the initial value for σ is:

(53) $$\begin{array}{c} {\hat{\sigma }}^{\left(0\right)}=\sqrt{\frac{\left(\frac{\hat{\mathrm{Var}[{\tilde{Y}}_{il}]}}{J}-\gamma_{0}^{2}\mu \right)}{\gamma_{0}^{2}+\mu_{0}^{2}}}, \end{array}$$

where $\hat{\mathrm{Var}[{\tilde{Y}}_{il}]}$ is an empirical estimate of the variance of ${\tilde{Y}}_{1}$.

**Bandwidth Selection:** A key issue in implementing the above method of estimation is the choice of the bandwidth. We express the bandwidth in the form $h_{B}=c_{B}s_{B}$, where $c_{B}\in \left\{0.3,0.4,0.5,0.7,0.9\right\}$, and $s_{B}$ is set equal to 1.48× median $\left(|{\tilde{Y}}_{il}-\mathrm{median}\left({\tilde{Y}}_{il}\right)|\right)/B$.

In all the tables below, we report the average (Ave), standard deviation (StD) and mean square error (MSE) to assess the performance of the proposed methods.

### 4.4. Analyses Without Contamination

From Table 2, Table 3, Table 4 and Table 5, we let true μ=2,σ=1, and take the kernel to be Gaussian kernel. In Table 2, we compare the estimates of the parameters as the dimension of the compressed data *S* increases. In this table, we allow *S* to take values in the set {1,2,5,10}. Also, we let the number of groups B=100, the bandwidth is chosen to be $c_{B}=0.3$, and $\gamma_{0}=0.1$. In addition, in Table 2, $S^{*}=1$ means that S=1 with $\gamma_{0}\equiv 0.$

From Table 2 we observe that as *S* increases, the estimates for μ and σ remain stable. The case $S^{*}=1$ is interesting, since even by storing the sum we are able to obtain point estimates which are close to the true value. In Table 3, we choose S=1,B=100 and $c_{B}=0.3$ and compare the estimates as $\gamma_{0}$ changes from 0.01 to 1.00. We can see that as $\gamma_{0}$ increases, the estimate for μ remains stable, whereas the bias, standard deviation and MSE for σ increase.

In Table 4, we fix S=1,B=100 and $\gamma_{0}=0.1$ and allow the bandwidth $c_{B}$ to increase. Also, $c_{B}^{*}=0.30$ means that the bandwidth is chosen as 0.30 with $\gamma_{0}\equiv 0.$ Notice that in this case when $c_{B}=0.9$ $B^{\frac{1}{2}}c_{B}=9$ while $B^{\frac{1}{2}}c_{B}^{2}=8.1$ which is not small as is required in assumption (**B2**). We notice again that as $c_{B}$ decreases, the estimates of μ and σ are close to the true value with small MSE and StD.

In Table 5, we let $S=1,c_{B}=0.3$ and $\gamma_{0}=0.1$ and let the number of groups *B* increase. This table implies that as *B* increases, the estimate performs better in terms of bias, standard deviation and MSE.

In Table 6, we set $\gamma_{0}\equiv 0$ and keep other settings same as Table 5. This table implies that as *B* increases, the estimate performs better in terms of bias, standard deviation and MSE. Furthermore, the standard deviation and MSE are slightly smaller than the results in Table 5.

We next move on to investigating the effect of other sensing variables. In the following table, we use Gamma model to generate the additive matrix $R_{l}$. Specifically, the mean of Gamma random variable is set as $\alpha_{0}\beta_{0}=1$, and the variance $var\equiv \alpha_{0}\beta_{0}^{2}$ is chosen from the set $\left\{0,0.01^{2},0.01,0.25,1.00\right\}$ which are also the variances in Table 3.

From Table 7, notice that using Gamma sensing variable yields similar results as Gaussian sensing variable. Our next example considers the case when the mean of the sensing variable is not equal to one and the sensing variable is taken to have a discrete distribution.Specifically, we use Bernoulli sensing variables with parameter *p*. Moreover, we fix S=1 and let pJ=S. Therefore p=1/J. Hence as *J* increases, the variance decreases. Now notice that in this case the mean of sensing variable is *p* instead of 1. In addition, $E[{\tilde{Y}}_{il}]=\mu$ and $\mathrm{Var}\left[{\tilde{Y}}_{il}\right]=\sigma^{2}+\mu^{2}\left(1-\frac{1}{J}\right)$. Hence we set the initial value as

$$\begin{array}{c} \begin{array}{c} \mu^{\left(0\right)}=\mathrm{median}\left({\tilde{Y}}_{il}\right),\\ \sigma^{\left(0\right)}=1.48\times \mathrm{median}\left(|{\tilde{Y}}_{il}-\mathrm{median}\left({\tilde{Y}}_{il}\right)|\right). \end{array} \end{array}$$

Additionally, we take B=100, $c_{B}=0.30$ and $s_{B}$ to be $1.48\times \mathrm{median}\left(|{\tilde{Y}}_{il}-\mathrm{median}\left({\tilde{Y}}_{il}\right)|\right).$

Table 8 shows that MHD method also performs well with Bernoulli sensing variable, although the bias of σ, standard deviation and mean squre error for both estimates are larger than those using Gaussian sensing variable and Gamma sensing variable.

### 4.5. Robustness and Model Misspecification

In this section, we provide a numerical assessment of the robustness of the proposed methodology. To this end, let

$$\begin{array}{c} f_{\alpha ,\eta }\left(x|\theta \right)=\left(1-\alpha \right)f\left(x|\theta \right)+\alpha \eta \left(x\right), \end{array}$$

where η(x) is a contaminating component, α∈[0,1). We generate the contaminated reduced data Y in the following way:

- Step 1. Generate $X_{l}$, where $X_{jl}\overset{i.i.d.}{∼}N\left(2,1\right)$.
- Step 2. Generate $R_{l}$, where $r_{ijl}\overset{i.i.d.}{∼}N\left(1,\gamma_{0}^{2}\right)$.
- Step 3. Generate uncontaminated ${\tilde{Y}}_{l}$ by calculating ${\tilde{Y}}_{l}=R_{l}X_{l}$.
- Step 4. Generate contaminated ${\tilde{Y}}_{il}^{c}$, where ${\tilde{Y}}_{il}^{c}={\tilde{Y}}_{il}+\eta \left(x\right)$ with probability α, and ${\tilde{Y}}_{il}^{c}={\tilde{Y}}_{il}$ with probability 1−α.

In the above description, the contamination with outliers is within blocks. A conceptual issue that one encounters is the meaning of outliers in this setting. Specifically, a data point which is an outlier in the original data set may not remain an outlier in the reduced data and vice-versa. Hence the concepts such as breakdown point and influence function need to be carefully studied. The tables below present one version of the robustness exhibited by the proposed method. In Table 9 and Table 10, we set $J=10^{4},B=100,S=1,\gamma_{0}=0.1,c_{B}=0.3,\eta =1000$. In addition, $\alpha^{*}=0$ means that α=0 with $\gamma_{0}\equiv 0.$

From the above Table we observe that, even under 50% contamination the estimate of the mean remains stable; however, the estimate of the variance is affected at high-levels of contamination (beyond 30%). An interesting and important issue is to investigate the role of $\gamma_{0}$ on the breakdown point of the estimator.

**Figure 2:**
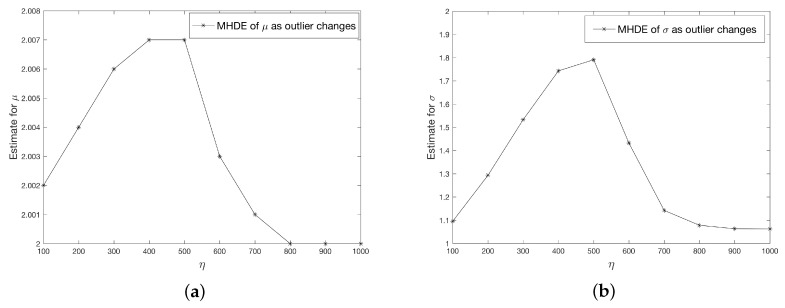
Comparison of estimates of μ (**a**) and σ (**b**) as outlier changes.

Finally, we investigate the bias in MHDE as a function of the values of the outlier. The graphs below (Figure 2) describe the changes to MHDE when outlier values (η) increase. Here we set $S=1,B=100,\gamma_{0}=0.1$. In addition, we let α=0.2, and η to take values from {100,200,300,400,500,600,700,800,900,1000}. We can see that as η increases, both $\hat{\mu }$ and $\hat{\sigma }$ increase up to η=500 then decrease, although $\hat{\mu }$ does not change too much. This phenomenon is because when the outlier value is small (or closer to the observations), then it may not be considered as an “outlier” by the MHD method. However, as the outlier values move “far enough” from other values, then the estimate for μ and σ remain the stable.

## 5. Example

**Figure 3:**
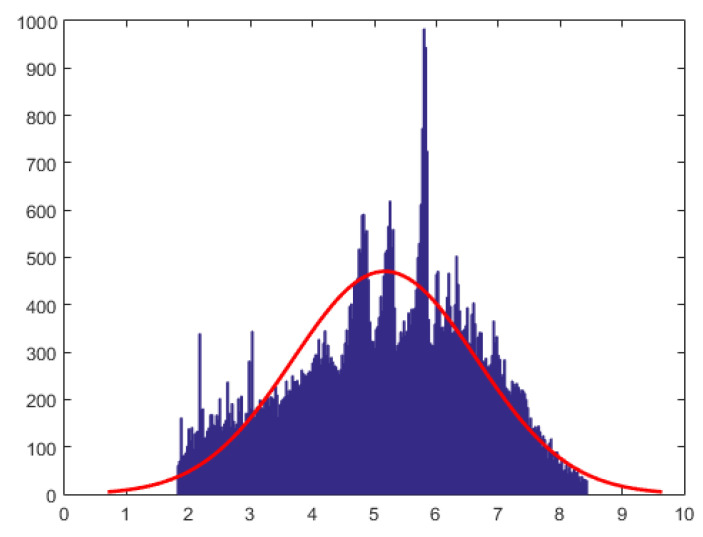
The histogram of credit payment data after Box-Cox transformation to Normality.

In this section we describe an analysis of data from financial analytics, using the proposed methods. The data are from a bank (a cash and credit card issuer) in Taiwan and the targets of analyses were credit card holders of the bank. The research focused on the case of customers’ default payments. The data set (see [27] for details) contains 180,000 observations and includes information on twenty five variables such as default payments, demographic factors, credit data, history of payment, and billing statements of credit card clients from April 2005 to September 2005. Ref. [28] study machine learning methods for evaluating the probability of default. Here, we work with the first three months of data containing 90,000 observations concerning bill payments. For our analyses we remove zero payments and negative payment from the data set and perform a logarithmic transformation of the bill payments . Since the log-transformed data was multi-modal and exhibited features of a mixture of normal distributions, we work with the log-transformed data with values in the range (6.1, 13). Next, we performed the Box-Cox transformation to the log-transformed data. This transformation identifies the best transformation that yields approximately normal distribution (which belongs to the location-scale family). Specifically, let L denote the log-transformed data in range (6.1, 13), then the data after Box-Cox transformation is given by $X=\left(L^{2}-1\right)/19.9091$. The histogram for X is given in Figure 3. The number of observations at the end of data processing was 70,000.

Our goal is to estimate the average bill payment during the first three months. For this, we will apply the proposed method. In this analysis, we assume that the target model for X is Gaussian and split the data, randomly, into B=100 blocks yielding J=700 observations per block.

In Table 11, “est” represents the estimator, “95% CI” stands for 95% confidence interval for the estimator. When analyzing the whole data and choosing bandwidth as $c_{n}=0.30$, we get the MHDE of μ to be $\hat{\mu }=5.183$ with 95% confidence interval (5.171,5.194), and the MHDE of σ as $\hat{\sigma }=1.425$ with confidence interval (1.418,1.433).

In Table 11, we choose the bandwidth as $c_{B}=0.30.$ Also, $S^{*}=1$ represents the case where S=1 and $\gamma_{0}\equiv 0$. In all other settings, we keep $\gamma_{0}=0.1$. We observe that all estimates are similar as *S* changes.

**Figure 4:**
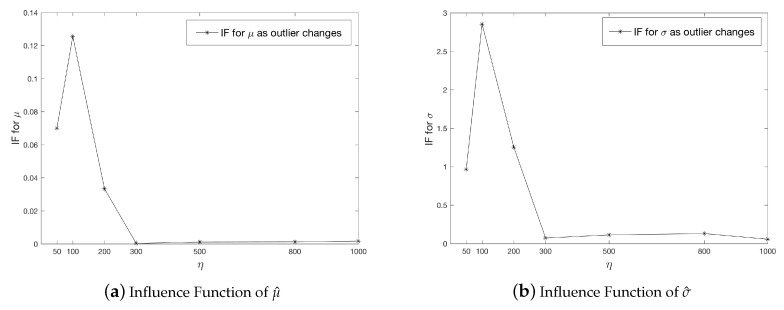
Influence Function of $\hat{\mu }$ (**a**) and $\hat{\sigma }$ (**b**) for MHDE.

Next we study the robustness of MHDE for this data by investigating the relative bias and studying the influence function. Specifically, we first reduce the dimension from J=700 to S=1 for each of the B=100 blocks and obtain the compressed data $\tilde{Y}$; next, we generate the contaminated reduced data ${\tilde{Y}}_{il}^{c}$ from step 4 in Section 4.5. Also, we set $\alpha =0.20,\gamma_{0}=0.20$; the kernel is taken to be to be Epanechnikov density with bandwidth $c_{B}=0.30$. η(x) is assumed to takes values in {50,100,200,300,500,800,1000} (note that the approximate mean of $\tilde{Y}$ is around 3600). Let $T_{\mathrm{MHD}}$ be the Hellinger distance functional. The influence function given by

$$\begin{array}{c} \begin{array}{c} \mathrm{IF}\left(\alpha ;T,\tilde{Y}\right)=\frac{T_{\mathrm{MHD}}\left({\tilde{Y}}^{c}\right)-T_{\mathrm{MHD}}\left(\tilde{Y}\right)}{\alpha }, \end{array} \end{array}$$

which we use to assess the robustness. The graphs shown below (Figure 4) illustrate how the influence function changes as the outlier values increase. We observe that for both estimates ($\hat{\mu }$ and $\hat{\sigma }$), the influence function first increase and then decrease fast. From η(x)=300, the influence functions remain stable and are close to zero, which clearly indicate that MHDE is stable.

**Additional Analyses:** The histogram in Figure 3 suggests that, may be a mixture of normal distributions may fit the log and Box-Cox transformed data better than the normal distribution. For this reason, we calculated the Hellinger distance between four component mixture (chosen using BIC criteria) and the normal distribution and this was determined to be 0.0237, approximately. Thus, the normal distribution (which belongs to the location-scale family) can be viewed as a misspecified target distribution; admittedly, one does lose information about the components of the mixture distribution due to model misspecification. However, since our goal was to estimate the overall mean and variance the proposed estimate seems to possess the properties described in the manuscript.

## 6. Discussion and Extensions

The results in the manuscript focus on the iterated limit theory for MHDE of the compressed data obtained from a location-scale family. Two pertinent questions arise: (i) is it easy to extend this theory to MHDE of compressed data arising from *non location-scale* family of distributions? and (ii) is it possible to extend the theory from iterated limits to a double limit? Turning to (i), we note that the heuristic for considering the location-scale family comes from the fact that the first and the second moment are consistently estimable for partially observed random walks (see [29,30]). This is related to the size of *J* and can be of exponential order. For such large *J*, other moments may not be consistently estimable. Hence, the entire theory goes through as long as one is considering parametric models f(·|θ), where $\theta =W(\mu ,\sigma^{2})$, for a known function W(·,·). The case in point is the Gamma distribution which can be re-parametrized in terms of the first two moments.

As for (ii), it is well-known that existence and equality of iterated limits for real sequences does not imply the existence of the double limit unless additional uniformity of convergence holds (see [31] for instance). Extension of this notion for distributional convergence requires additional assumptions and are investigated in a different manuscript wherein more general divergences are also considered.

## 7. Concluding Remarks

In this paper we proposed the Hellinger distance-based method to obtain robust estimates for mean and variance in a location-scale model using compressed data. Our extensive theoretical investigations and simulations show the usefulness of the methodology and hence can be applied in a variety of scientific settings. Several theoretical and practical questions concerning robustness in a big data setting arise. For instance, the effect of the variability in the R matrix and its effect on outliers are important issues that need further investigation. Furthermore, statistical properties such as uniform consistency and uniform asymptotic normality under different choices for the distribution of R would be useful. These are under investigation by the authors.

## Figures and Tables

**Table 1:** Illustration of Data Reduction Mechanism, Here $r_{il}^{*}=\left(r_{i\cdot l},\omega_{il}\right)$.

	Grp 1	Grp 2	⋯	Grp B		Grp 1	Grp 2	⋯	Grp B
Original	$X_{11}$	$X_{12}$	⋯	$X_{1B}$	Compressed	$\left({\tilde{Y}}_{11},r_{11}^{*}\right)$	$\left({\tilde{Y}}_{12},r_{12}^{*}\right)$	⋯	$\left({\tilde{Y}}_{1B},r_{1B}^{*}\right)$
Data	$X_{21}$	$X_{22}$	⋯	$X_{2B}$	Data	$\left({\tilde{Y}}_{21},r_{21}^{*}\right)$	$\left({\tilde{Y}}_{22},r_{22}^{*}\right)$	⋯	$\left({\tilde{Y}}_{2B},r_{2B}^{*}\right)$
	⋮	⋮	⋮	⋮	$\overset{S\ll J}{⟹}$	⋮	⋮	⋮	⋮
	$X_{J1}$	$X_{J2}$	⋯	$X_{JB}$		$\left({\tilde{Y}}_{S1},r_{S1}^{*}\right)$	$\left({\tilde{Y}}_{S2},r_{S2}^{*}\right)$	⋯	$\left({\tilde{Y}}_{SB},r_{SB}^{*}\right)$

**Table 2:** MHDE as the dimension *S* changes for compressed data $\tilde{Y}$ using Gaussian kernel.

	$\hat{\mu }$			$\hat{\sigma }$		
	Ave	StD $\times 10^{3}$	MSE $\times 10^{3}$	Ave	StD $\times 10^{3}$	MSE $\times 10^{3}$
$S^{*}=1$	2.000	1.010	0.001	1.016	74.03	5.722
S=1	2.000	1.014	0.001	1.018	74.22	5.844
S=2	2.000	1.005	0.001	1.019	73.81	5.832
S=5	2.000	0.987	0.001	1.017	74.16	5.798
S=10	2.000	0.995	0.001	1.019	71.87	5.525

**Table 3:** MHDE as $\gamma_{0}$ changes for compressed data $\tilde{Y}$ using Gaussian kernel.

	$\hat{\mu }$			$\hat{\sigma }$		
	Ave	StD $\times 10^{3}$	MSE $\times 10^{3}$	Ave	StD $\times 10^{3}$	MSE $\times 10^{3}$
$\gamma_{0}=0.00$	2.000	1.010	0.001	1.016	74.03	5.722
$\gamma_{0}=0.01$	2.000	1.017	0.001	1.015	74.83	5.814
$\gamma_{0}=0.10$	2.000	1.023	0.001	1.021	72.80	5.717
$\gamma_{0}=0.50$	2.000	1.119	0.001	1.076	72.59	11.08
$\gamma_{0}=1.00$	2.000	1.399	0.002	1.226	82.21	57.75

**Table 4:** MHDE as the bandwidth $c_{B}$ changes for compressed data $\tilde{Y}$ using Gaussian kernel.

	$\hat{\mu }$			$\hat{\sigma }$		
	Ave	StD $\times 10^{3}$	MSE $\times 10^{3}$	Ave	StD $\times 10^{3}$	MSE $\times 10^{3}$
$c_{B}^{*}=0.30$	2.000	1.010	0.001	1.016	74.03	5.722
$c_{B}=0.30$	2.000	1.014	0.001	1.018	74.22	5.844
$c_{B}=0.40$	2.000	1.015	0.001	1.063	79.68	10.26
$c_{B}=0.50$	2.000	1.014	0.001	1.108	82.33	18.33
$c_{B}=0.70$	2.000	1.004	0.001	1.212	93.96	53.64
$c_{B}=0.90$	2.000	1.009	0.001	1.346	110.5	132.2

**Table 5:** MHDE as *B* changes for compressed data $\tilde{Y}$ using Gaussian kernel with $\gamma_{0}=0.1$.

	$\hat{\mu }$			$\hat{\sigma }$		
	Ave	StD $\times 10^{3}$	MSE $\times 10^{3}$	Ave	StD $\times 10^{3}$	MSE $\times 10^{3}$
B=20	2.000	2.205	0.005	1.739	378.5	688.6
B=50	2.000	1.409	0.002	1.136	125.2	34.17
B=100	2.000	1.010	0.001	1.016	74.03	5.722
B=500	2.000	0.455	0.000	0.972	32.63	1.873

**Table 6:** MHDE as *B* changes for compressed data $\tilde{Y}$ using Gaussian kernel with $\gamma_{0}=0$.

	$\hat{\mu }$			$\hat{\sigma }$		
	Ave	StD $\times 10^{3}$	MSE $\times 10^{3}$	Ave	StD $\times 10^{3}$	MSE $\times 10^{3}$
B=20	2.000	2.282	0.005	1.749	381.4	706.0
B=50	2.000	1.440	0.002	1.148	125.2	37.42
B=100	2.000	1.014	0.001	1.018	74.22	5.844
B=500	2.000	0.465	0.000	0.973	31.33	1.692

**Table 7:** MHDE as variance changes for compressed data $\tilde{Y}$ using Gaussian kernel under Gamma sensing variable.

	$\hat{\mu }$			$\hat{\sigma }$		
	Ave	StD $\times 10^{3}$	MSE $\times 10^{3}$	Ave	StD $\times 10^{3}$	MSE $\times 10^{3}$
var=0.00	2.000	1.010	0.001	1.016	74.03	5.722
$var=0.01^{2}$	2.000	1.005	0.001	1.016	74.56	5.806
var=0.01	2.000	1.006	0.001	1.018	73.70	5.762
var=0.25	2.000	1.120	0.001	1.078	73.70	11.56
var=1.00	2.000	1.438	0.001	1.228	81.94	58.48

**Table 8:** MHDE as *J* changes for compressed data $\tilde{Y}$ using Gaussian kernel under Bernoulli sensing variable.

	$\hat{\mu }$			$\hat{\sigma }$		
	Ave	StD $\times 10^{3}$	MSE $\times 10^{3}$	Ave	StD $\times 10^{3}$	MSE $\times 10^{3}$
J=10	2.000	104.9	11.01	1.215	97.78	55.79
J=100	1.998	104.5	10.93	1.201	104.5	51.26
J=1000	1.998	104.7	10.96	1.195	106.6	49.36
J=5000	2.001	103.9	10.80	1.200	105.7	51.20
J=10000	1.996	105.1	11.07	1.196	104.4	49.16

**Table 9:** MHDE as α changes for contaminated data $\tilde{Y}$ using Gaussian kernel.

	$\hat{\mu }$			$\hat{\sigma }$		
	Ave	StD $\times 10^{3}$	MSE $\times 10^{3}$	Ave	StD $\times 10^{3}$	MSE $\times 10^{3}$
$\alpha^{*}=0.00$	2.000	1.010	0.001	1.016	74.03	5.722
α=0.00	2.000	1.014	0.001	1.018	74.22	5.844
α=0.01	2.000	1.002	0.001	1.022	74.89	6.079
α=0.05	2.000	1.053	0.001	1.023	77.86	6.599
α=0.10	2.000	1.086	0.001	1.034	79.30	7.350
α=0.20	2.000	1.146	0.001	1.073	93.45	14.06
α=0.30	2.001	7.205	0.054	1.264	688.2	542.5
α=0.40	2.026	21.60	1.100	3.454	1861	9480
α=0.50	2.051	14.00	2.600	4.809	1005	15513

**Table 10:** MHDE as α changes for contaminated data $\tilde{Y}$ using Epanechnikov kernel.

	$\hat{\mu }$			$\hat{\sigma }$		
	Ave	StD $\times 10^{3}$	MSE $\times 10^{3}$	Ave	StD $\times 10^{3}$	MSE $\times 10^{3}$
$\alpha^{*}=0.00$	2.000	0.972	0.001	1.008	73.22	5.425
α=0.00	2.000	1.014	0.001	1.018	74.22	5.844
α=0.01	2.000	0.978	0.001	1.028	107.4	12.19
α=0.05	2.000	1.264	0.002	1.025	108.7	12.35
α=0.10	2.000	1.202	0.001	1.008	114.7	13.09
α=0.20	2.000	1.263	0.002	1.046	129.8	18.76
α=0.30	2.001	5.098	0.026	1.104	557.8	318.9
α=0.40	2.021	21.80	0.900	3.004	1973	7870
α=0.50	2.051	10.21	3.000	4.893	720.4	15669

**Table 11:** MHDE from the real data analysis.

		$\hat{\mu }$	$\hat{\sigma }$
$S^{*}=1$	est	5.171	1.362
	95% CI	(4.904, 5.438)	(1.158, 1.540)
S=1	est	5.171	1.391
	95% CI	(4.898, 5.443)	(1.183, 1.572)
S=5	est	5.172	1.359
	95% CI	(4.905, 5.438)	(1.155, 1.535)
S=10	est	5.171	1.372
	95% CI	(4.902, 5.440)	(1.167, 1.551)
S=20	est	5.171	1.388
	95% CI	(4.899, 5.443)	(1.180, 1.569)

## Abbreviations

The following abbreviations are used in this manuscript:

MHDE	Minimum Hellinger Distance Estimator
MHD	Minimum Hellinger Distance
i.i.d.	independent and identically distributed
MLE	Maximum Likelihood Estimator
CI	Confidence Interval
IF	Influence Function
RHS	Right Hand Side
LHS	Left Hand Side
BFGS	Broyden-Fletcher-Goldfarb-Shanno
var	Variance
StD	Standard Deviation
MSE	Mean Square Error
